# Synthesis and Evaluation of Small Molecule Drug Conjugates Harnessing Thioester-Linked Maytansinoids

**DOI:** 10.3390/pharmaceutics14071316

**Published:** 2022-06-21

**Authors:** Chen-Fu Lo, Tai-Yu Chiu, Yu-Tzu Liu, Li-Rung Huang, Teng-Kuang Yeh, Kuan-Hsun Huang, Kuan-Liang Liu, Chia-Yu Hsu, Ming-Yu Fang, Yu-Chen Huang, Tsu-An Hsu, Chiung-Tong Chen, Lun Kelvin Tsou

**Affiliations:** 1 Institute of Biotechnology and Pharmaceutical Research, National Health Research Institutes, Miaoli 35053, Taiwan; chenfu10@nhri.edu.tw (C.-F.L.); taiyu.chiu@nhri.edu.tw (T.-Y.C.); tkyeh@nhri.edu.tw (T.-K.Y.); geniuscan2003@gmail.com (K.-H.H.); tedliu@nhri.edu.tw (K.-L.L.); chiayu85@gmail.com (C.-Y.H.); fnfn@nhri.edu.tw (M.-Y.F.); 960626@nhri.edu.tw (Y.-C.H.); tsuanhsu@nhri.edu.tw (T.-A.H.); ctchen@nhri.edu.tw (C.-T.C.); 2 Institute of Molecular and Genomic Medicine, National Health Research Institutes, Miaoli 35053, Taiwan; yuki1225@nhri.edu.tw (Y.-T.L.); lrhuang@nhri.edu.tw (L.-R.H.)

**Keywords:** small molecule drug conjugates, zinc(II)-dipicolylamine, phosphatidylserine, thioester, maytansinoids

## Abstract

Ligand-targeting drug conjugates are a class of clinically validated biopharmaceutical drugs constructed by conjugating cytotoxic drugs with specific disease antigen targeting ligands through appropriate linkers. The integrated linker-drug motif embedded within such a system can prevent the premature release during systemic circulation, thereby allowing the targeting ligand to engage with the disease antigen and selective accumulation. We have designed and synthesized new thioester-linked maytansinoid conjugates. By performing in vitro cytotoxicity, targeting ligand binding assay, and in vivo pharmacokinetic studies, we investigated the utility of this new linker-drug moiety in the small molecule drug conjugate (SMDC) system. In particular, we conjugated the thioester-linked maytansinoids to the phosphatidylserine-targeting small molecule zinc dipicolylamine and showed that **Zn8_DM1** induced tumor regression in the HCC1806 triple-negative breast cancer xenograft model. Moreover, in a spontaneous sorafenib-resistant liver cancer model, **Zn8_DM1** exhibited potent antitumor growth efficacy. From quantitative mRNA analysis of **Zn8_DM1** treated-tumor tissues, we observed the elevation of gene expressions associated with a “hot inflamed tumor” state. With the identification and validation of a plethora of cancer-associated antigens in the “omics” era, this work provided the insight that antibody- or small molecule-based targeting ligands can be conjugated similarly to generate new ligand-targeting drug conjugates.

## 1. Introduction

The rapid development of drug conjugates with antigen targeting ligands in cancer treatment has gained particular attention in the past decade [1,2]. Ligand-targeted drug delivery consists of three main components; a targeting ligand that provides selective association with the disease antigen, a linker harnessing a controlled release functional group, and a cytotoxic drug [2,3,4]. As the result of more efficient and better conjugation strategies between the cytotoxic payloads, linkers, and the targeting ligands, modular design was employed. Many antibody-drug conjugates (ADC) were prepared, and several have met with success for the treatment of cancers in the clinical setting [1,5,6,7]. Concurrently, a drug delivery platform that employs small organic molecules as a targeting moiety has emerged as an alternative strategy [3,8,9]. The development of small molecule drug conjugate (SMDC) allows manageable synthesis in process development and pharmacokinetics optimizations compared to ADC [8,9]. In addition to being less immunogenic in nature, the SMDC’s smaller molecular weights might translate to better solid tumor penetrating properties as various SMDCs have been studied in preclinical and clinical settings for the treatment of cancers [8,9]. Nonetheless, integrated linker design in both ADC and SMDC governs the conjugate’s circulation stability in vivo to prevent premature release of the cytotoxic payload that can render diminished efficacy and unfavorable toxicity profiles [10,11]. Clinically validated linkers, such as protease cathepsin-sensitive valine-citrulline (Val-Cit) or phenylalanine-lysine (Phe-Lys) dipeptides for controlled release of monomethyl auristatin E (MMAE) and pH-sensitive hydrazine linkers with calicheamicin were implemented to confer stability of ADCs during systemic circulation and facilitate the release of the cytotoxic drugs within cancer cells [10,11]. Moreover, linkers containing stable disulfide bonds can be advantageous with high intracellular or tumor microenvironment (TME) concentrations of reductive glutathione to liberate the payload, which might confer bystander killing of heterogeneous tumors [12]. With identifications of new cytotoxic payloads, utilities of new cleavable linkers have also been investigated [13,14,15].

In the conjugated form, potent tubulin inhibitors are validated cytotoxic payloads in clinical settings since the delivery platform overcomes the lack of tumor specificity of the chemotherapeutics administered without the targeting ligand. In particular, Maytansine-analog DM1 and Dolastatin-analog MMAE are potent tubulin inhibitors in the ADCs that have met with clinical success [12]. For example, ADCs with disulfide-linkages or thioether-linked DM1 conjugates showed increased half-life and in vivo stability [16,17]. However, thioester-linked maytansinoids are primarily underexplored. We previously showed that the bulky cyclohexyl-para-chlorophenyl moiety in the linker could provide conformational hindrance for 4-(2-aminoethyl)-benzenesulfonyl fluoride hydrochloride (AEBSF)-sensitive serine proteases-mediated release of the cytotoxic payload [18]. Since thioesters were substrates of serine proteases [19,20], the linker that harnessed a cyclohexyl-para-chlorophenyl moiety conjugating with a maytansinoid via a thioester bond thus warrants investigation in a chemically-defined small molecule drug conjugate. Notably, phosphatidylserine (PS) has been reported as a tumor-associating antigen [21,22,23,24]. Zinc(II)-dipicolylamine (ZnDPA) equipped with a near infra-red fluorophore or radiolabeled Zn-DPA complexes were shown to specifically accumulate in a variety of xenografted tumors through their interaction with PS [25]. Herein, we report a facile synthetic strategy to prepare thioester-linked maytansinoids and to conjugate them with ZnDPA. We then profiled the conjugates’ cytotoxicities, in vivo stabilities, and antitumor efficacies to demonstrate the utilities of the newly constructed thioester-linker maytansinoids. 

## 2. Experimental Section

### 2.1. Materials and Methods

**Synthesis. General**. Unless otherwise stated, all materials were obtained from the market and used as specified. Reactions were carried out under argon or nitrogen and monitored by analytical thin-layer chromatography using glass-backed plates (5 × 10 cm) pre-coated with 60 F254 silica gel (supplied by Merck & Co., Inc., Whitehouse Station in Readington Township, NJ, USA). Flame-dried glassware was cooled and used in an argon or nitrogen atmosphere for reactions requiring anhydrous conditions. The resulting chromatograms were observed with a UV lamp (λ = 254 nm) and then charred with a heat gun and immersed in an ethanol solution containing sulphuric acid (3% *v*/*v*) or vanillin (5% *w*/*v*) with phosphomolybdic acid (2.5% *w*/*v*). Solvents used for the reaction included THF, diethyl ether (ether), DMF, toluene, dichloromethane, and pyridine, which were dried and distilled under argon or nitrogen before use. Flash chromatography was routinely used to separate and purify product mixtures using silica gel 60 in sizes 230–400 mesh supplied by Merck, with elution systems given in volume/volume ratios. ^1^H and ^13^C NMR spectroscopy was performed by Varian Mercury-300 (300 MHz), Varian Mercury-400 (400 MHz), Bruker Avance Neo AV4400, AV4600, and DMX-600 (600 MHz) collections; chemical shift values are reported in parts per million (δ) units relative to TMS. Multiples are expressed as s (singlet), br s (generalized singlet), d (doublet), t (triplet), q (quadruplet), and dd (doublet of doublet), dt (doublet of triplet), and m (multiplet). Coupling constants (J) are reported in hertz. Using an Agilent 1100 MSD mass spectrometer, electrospray mass spectra (ESMS) were recorded as m/z values, and to obtain HRMS, a Bruker (Impact HD, Tokyo, Japan) Autoflex Max TOF/TOF (MALDI) was used. All compounds with >95% purity were determined by an Agilent 1100 series HPLC system using a C18 column (Thermo Golden, 4.6 mm × 250 mm); see Supplementary Materials for detailed conditions. The IUPAC nomenclature of the compounds was determined using ACD/Name Pro software.

### 2.2. 4-(2-(1-(4-Chlorophenyl)cyclohexane-1-carbonyl)-14,14-dimethyl-12-oxo-5,8,13-trioxa-2,11-diazapentadecyl)benzoic acid (***2***)

To compound 1 (300 mg, 0.48 mmol) in 100 mL MeOH, 10 mL 0.5M LiOH_(aq)_ was added. The reaction mixture was stirred at room temperature for 15 h. The solvent was removed and the residue was redissolved in 100 mL CH_2_Cl_2_. The insoluble residue was then filtered off. The filtrate was washed with CH_2_Cl_2_, dried over Na_2_SO_4(s)_ and the solvent was removed under vacuum. The product was obtained as white powder (compound **2**, 263 mg, 0.43 mmol, 90%). ^1^H NMR (400 MHz, CD_3_OD) δ 7.86 (d, *J* = 7.4 Hz, 2H), 7.35 (d, *J* = 8.8 Hz, 2H), 7.30 (d, *J* = 8.8 Hz, 2H), 6.93 (br, 2H), 4.31 (br, 2H), 3.64 (d, *J* = 13.6 Hz, 2H), 3.56–3.39 (m, 6H), 3.19 (t, *J* = 5.7 Hz, 2H), 2.31 (br, 2H), 1.95–1.52 (m, 8H), 1.40 (s, 9H), 1.30 (br, 2H). ^13^C NMR (101 MHz, CD_3_OD) δ 177.04, 174.94, 158.40, 146.06, 140.22, 138.33, 133.51, 130.62, 130.19, 128.19, 127.02, 80.09, 71.40, 71.05, 69.70, 53.75, 52.49, 47.32, 41.28, 38.00, 28.76, 26.97, 24.96. HRMS(ESI) calculated for C_32_H_44_ClN_2_O_7_^+^: 603.2832, found: 603.2827 (M + H^+^)^+^.

### 2.3. (14R,16R,33R,2S,4S,10E,12Z,14S)-86-Chloro-14-hydroxy-85,14-dimethoxy-33,2,7,10-tetramethyl-12,6-dioxo-7-aza-1(6,4)-oxazinana-3(2,3)-oxirana-8(1,3)-benzenacyclotetradecaphane-10,12-dien-4-yl N-(3-((4-(2-(1-(4-chlorophenyl)cyclohexane-1-carbonyl)-14,14-dimethyl-12-oxo-5,8,13-trioxa-2,11-diazapentadecyl)benzoyl)thio)propanoyl)-N-methyl-L-alaninate (***3***)

To a stirred solution of compound 2 (150 mg, 0.24 mmol) dissolved in DMF (20 mL), (14S,16S,32R,33S,2R,4S,10E,12E,14R)-86-chloro-14-hydroxy-85,14-dimethoxy-33,2,7,10-tetramethyl-12,6-dioxo-7-aza-1(6,4)-oxazinana-3(2,3)-oxirana-8(1,3)-benzenacyclotetradecaphane-10,12-dien-4-yl N-(3-mercaptopropanoyl)-N-methyl-D-alaninate (DM-1) (100 mg, 0.13 mmol), 4-Dimethylaminopyridine (DMAP, 30 mg, 0.24 mmol), and *N*-(3-Dimethylaminopropyl)-*N*′-ethylcarbodiimide hydrochloride (EDCl, 30 mg, 0.18 mmol) were added. The mixture was stirred at room temperature in the dark for 15 h. After completion of the reaction, the mixture was extracted with CH_2_Cl_2_ (100 mL) and saturated aqueous NaHCO_3_ (100 mL) and H_2_O (4 × 100 mL). The combined organic layers were dried over MgSO_4_, concentrated under reduced pressure. Purification of the crude residue by reversed-phase chromatography on Lichroprep@RP-18 was eluted with ACN gradient grade to get compound 3 (70 mg, 0.05 mmol, 40%). ^1^H NMR (600 MHz, CDCl_3_) δ 7.60 (s, 2H), 7.29 (d, *J* = 8.6 Hz, 2H), 7.20 (d, *J* = 8.7 Hz, 2H), 6.91 (s, 1H), 6.77 (d, *J* = 11.3 Hz, 2H), 6.66 (s, 1H), 6.44 (dd, *J* = 15.2, 10.9 Hz, 1H), 6.23 (s, 1H), 5.63 (dd, *J* = 15.6, 9.2 Hz, 1H), 5.44 (q, *J* = 6.7 Hz, 1H), 4.89 (s, 1H), 4.73 (dd, *J* = 12.1, 3.1 Hz, 1H), 4.33–4.18 (m, 2H), 3.95 (s, 3H), 3.77 (d, *J* = 12.9 Hz, 1H), 3.67 (s, 1H), 3.59–3.40 (m, 7H), 3.40–3.30 (m, 5H), 3.30–3.22 (m, 3H), 3.16–3.03 (m, 5H), 3.02 (d, *J* = 9.7 Hz, 1H), 2.81 (s, 4H), 2.69 (d, *J* = 6.3 Hz, 1H), 2.57 (dd, *J* = 14.4, 12.1 Hz, 1H), 2.34 (t, *J* = 7.0 Hz, 1H), 2.27–2.12 (m, 3H), 1.69 (s, 10H), 1.54 (d, *J* = 15.3 Hz, 1H), 1.41 (s, 10H), 1.29 (dd, *J* = 10.4, 6.6 Hz, 6H), 1.26–1.19 (m, 2H), 0.79 (s, 3H). ^13^C NMR (101 MHz, CDCl_3_) δ 170.90, 170.82, 168.85, 156.06, 155.88, 152.31, 142.09, 141.03, 139.70, 133.54, 132.47, 129.18, 127.54, 127.38, 126.93, 126.51, 125.33, 122.86, 118.73, 113.18, 88.57, 80.99, 79.44, 78.39, 74.24, 70.44, 70.36, 69.00, 67.47, 60.00, 56.76, 56.66, 52.45, 51.19, 46.69, 40.45, 38.99, 37.29, 36.20, 35.34, 34.18, 32.46, 30.63, 28.53, 25.95, 24.28, 23.77, 15.68, 14.70, 13.46, 12.13, 0.13. HRMS(ESI) calculated for C_67_H_89_Cl_2_N_5_O_16_S^+^: 1322.5475, found: 1322.5461 (M + H^+^)^+^.

### 2.4. (14R,16R,33R,2S,4S,10E,12Z,14S)-86-Chloro-14-hydroxy-85,14-dimethoxy-33,2,7,10-tetramethyl-12,6-dioxo-7-aza-1(6,4)-oxazinana-3(2,3)-oxirana-8(1,3)-benzenacyclotetradecaphane-10,12-dien-4-yl N-(4-((4-(2-(1-(4-chlorophenyl)cyclohexane-1-carbonyl)-14,14-dimethyl-12-oxo-5,8,13-trioxa-2,11-diazapentadecyl)benzoyl)thio)-4-methylpentanoyl)-N-methyl-D-alaninate (***4***)

To a stirred solution of compound 2 (150 mg, 0.24 mmol) dissolved in DMF (20 mL), (14R,16R,33R,2S,4S,10E,12Z,14S)-86-chloro-14-hydroxy-85,14-dimethoxy-33,2,7,10-tetramethyl-12,6-dioxo-7-aza-1(6,4)-oxazinana-3(2,3)-oxirana-8(1,3)-benzenacyclotetradecaphane-10,12-dien-4-yl *N*-(4-mercapto-4-methylpentanoyl)-N-methyl-D-alaninate (DM-4) (110 mg, 0.14 mmol) and 4-Dimethylaminopyridine (DMAP, 30 mg, 0.24 mmol) and *N*-(3-Dimethylaminopropyl)-*N*´-ethylcarbodiimide hydrochloride (EDCl, 30 mg, 0.18 mmol) were added. The mixture was stirred at room temperature in the dark for 15 h. After completion of the reaction, the mixture was extracted with CH_2_Cl_2_ (100 mL) and saturated aqueous NaHCO_3_ (100 mL) and H_2_O (4 × 100 mL). The combined organic layers were dried over MgSO_4_, and concentrated under reduced pressure. Purification of the crude residue by reversed-phase chromatography on Lichroprep@RP-18 was eluted with ACN gradient grade to get compound 4 (55 mg, 0.04 mmol, 28%). ^1^H NMR (600 MHz, CDCl_3_) δ 7.52 (s, 2H), 7.30 (d, *J* = 8.7 Hz, 2H), 7.21 (d, *J* = 8.7 Hz, 2H), 6.91 (s, 1H), 6.80 (s, 1H), 6.76 (d, *J* = 11.1 Hz, 1H), 6.53 (s, 1H), 6.41 (dd, *J* = 15.4, 11.1 Hz, 1H), 6.24 (s, 1H), 5.69 (dd, *J* = 15.3, 9.0 Hz, 1H), 5.41 (q, *J* = 6.6 Hz, 1H), 4.91 (s, 1H), 4.74 (dd, *J* = 12.1, 3.1 Hz, 1H), 4.25 (t, *J* = 12.4 Hz, 2H), 3.95 (s, 2H), 3.66 (d, *J* = 13.0 Hz, 2H), 3.57–3.41 (m, 7H), 3.35 (s, 4H), 3.32–3.24 (m, 3H), 3.15 (d, *J* = 11.3 Hz, 1H), 3.03 (s, 2H), 2.96 (s, 2H), 2.80 (s, 3H), 2.57–2.45 (m, 2H), 2.38 (t, *J* = 12.3 Hz, 2H), 2.25–2.08 (m, 3H), 1.70 (s, 10H), 1.53 (s, 5H), 1.47–1.35 (m, 12H), 1.35–1.22 (m, 10H), 0.96 (d, *J* = 6.6 Hz, 1H), 0.91–0.81 (m, 3H), 0.77 (s, 3H). ^13^C NMR (101 MHz, CDCl_3_) δ 175.12, 172.01, 171.11, 168.81, 156.07, 155.83, 152.35, 141.99, 141.48, 139.23, 136.52, 133.49, 132.47, 129.19, 127.74, 127.04, 126.45, 125.54, 122.18, 118.57, 113.15, 88.53, 81.00, 79.40, 78.18, 74.18, 70.43, 70.37, 68.97, 67.36, 60.03, 56.73, 56.55, 52.43, 51.12, 46.82, 40.46, 38.90, 37.17, 36.22, 36.06, 35.17, 32.37, 30.74, 30.08, 29.45, 28.87, 28.54, 28.06, 25.96, 23.77, 15.58, 14.66, 13.35, 12.18, 0.13. HRMS(ESI) calculated for C_70_H_95_Cl_2_N_5_NaO_16_S^+^: 1364.5944, found: 1364.5928 (M+ H^+^)^+^.

### 2.5. 4-(12-(4-((N-(4-(3,5-bis((bis(pyridin-2-ylmethyl)amino)methyl)phenoxy)butyl)-1-(4-chlorophenyl)cyclohexane-1-carboxamido)methyl)phenyl)-2-(1-(4-chlorophenyl)cyclohexane-1-carbonyl)-12-oxo-5,8-dioxa-2,11-diazadodecyl)benzoic acid (***6***)

(a) To compound 1 (1.2 g, 1.94 mmol) in 50 mL CH_2_Cl_2_, 2 mL of TFA was added. The reaction mixture was stirred at room temperature for 3 h. The NaHCO_3_(aq.) solution was added and then extracted with 100 mL CH_2_Cl_2_. The combined organic extracts were washed with brine, dried over Na_2_SO_4(s)_, filtered, and evaporated. The product was obtained as oil (methyl 4-((N-(2-(2-(2-aminoethoxy)ethoxy)ethyl)-1-(4-chlorophenyl)cyclohexane-1-carboxamido)methyl)benzoate, 0.96g, 1.85 mmol, 95%). (b) A solution of compound 5 (1.16 g, 1.23 mmol) in 10 mL DMF was heated to 40 °C. 1-Ethyl-3-(3-dimethylaminopropyl)carbodiimide (EDCI, 0.2 g, 1.28 mmol) and hydroxybenzotriazole (HOBt, 0.2g, 1.48 mmol) were added and the reaction was allowed to stir at 40 °C for 30 min. Methyl 4-((N-(2-(2-(2-aminoethoxy)ethoxy)ethyl)-1-(4-chlorophenyl)cyclohexane-1-carboxamido)methyl)benzoate (0.96 g, 1.85 mmol) was added followed by addition of N-methylmorpholine (NMM, 0.5ml, 4.5mmol). The reaction was stirred at 40 °C for 15 h, after which time it was diluted with H_2_O. The aqueous solution was separated and extracted with 200 mL of CH_2_Cl_2_. The combined organic extracts were washed with brine, dried over Na_2_SO_4(s)_, filtered, and evaporated. Purification of the crude residue by flash chromatography on pH = 7 silica gel eluting with MeOH/CH_2_Cl_2_ (1:9) gave rise to ester compounds (0.71 g, 0.49 mmol, 40%). (c) Then, to ester compounds (0.71 g, 0.49 mmol) in 100 mL of MeOH, 10 mL of 0.5M LiOH_(aq)_ was added. The reaction mixture was stirred at room temperature for 15 h. The solvent was removed and the residue was redissolved in 100 mL CH_2_Cl_2_. The insoluble residue was then filtered off. The filtrate was dried over Na_2_SO_4(s)_ and the solvent removed under vacuum. The product was obtained as white powder (compound 6, 0.58 g, 0.41 mmol, 82%). ^1^H NMR (300 MHz, CDCl_3_) δ 8.55 (d, *J* = 4.9 Hz, 4H), 7.93 (s, 1H), 7.73–7.45 (m, 12H), 7.25–7.09 (m, 12H), 7.04–6.56 (m, 6H), 4.12 (q, *J* = 7.1 Hz, 1H), 3.81 (s, 11H), 3.73–3.50 (m, 17H), 3.48 (s, 1H), 3.34 (br, 2H), 3.05 (br, 3H), 2.97–2.41 (m, 9H), 2.21 (br, 5H), 2.04 (s, 1H), 1.25 (t, *J* = 7.1 Hz, 5H). ^13^C NMR (101 MHz, CD_3_OD) δ 161.01, 149.88, 146.48, 141.99, 139.11, 133.90, 131.04, 130.59, 129.00, 128.61, 128.11, 127.47, 125.08, 124.24, 115.38, 71.84, 71.01, 70.06, 61.29, 60.24, 54.11, 52.85, 41.41, 38.36, 27.67, 27.36, 25.35. HRMS(ESI) calculated for C_84_H_94_Cl_2_N_9_O_8_^+^: 1426.6602, found: 1426.6613(M + H^+^)^+^.

### 2.6. (14R,16R,33R,2S,4S,10E,12Z,14S)-86-Chloro-14-hydroxy-85,14-dimethoxy-33,2,7,10-tetramethyl-12,6-dioxo-7-aza-1(6,4)-oxazinana-3(2,3)-oxirana-8(1,3)-benzenacyclotetradecaphane-10,12-dien-4-yl N-(3-((4-(12-(4-((N-(4-(3,5-bis((bis(pyridin-2-ylmethyl)amino)methyl)phenoxy)butyl)-1-(4-chlorophenyl)cyclohexane-1-carboxamido)methyl)phenyl)-2-(1-(4-chlorophenyl)cyclohexane-1-carbonyl)-12-oxo-5,8-dioxa-2,11-diazadodecyl)benzoyl)thio)propanoyl)-N-methyl-L-alaninate (***7***)

To a stirred solution of compound 6 (100 mg, 0.070 mmol) dissolved in DMF (3 mL), (14S,16S,32R,33S,2R,4S,10E,12E,14R)-86-chloro-14-hydroxy-85,14-dimethoxy-33,2,7,10-tetramethyl-12,6-dioxo-7-aza-1(6,4)-oxazinana-3(2,3)-oxirana-8(1,3)-benzenacyclotetradecaphane-10,12-dien-4-yl N-(3-mercaptopropanoyl)-N-methyl-D-alaninate (DM-1) (60 mg, 0.081 mmol, 1.1 equiv.), 4-dimethylaminopyridine (DMAP, 15 mg, 0.122 mmol, 1.7 equiv.) and *N*-(3-dimethylaminopropyl)-*N*′-ethylcarbodiimide hydrochloride (EDCl, 15 mg, 0.096 mmol, 1.3 equiv.) were added. The mixture was stirred at room temperature in the dark for 15 h. After completion of the reaction, the mixture was extracted with CH_2_Cl_2_ (100 mL), saturated aqueous NaHCO_3_ (100 mL), and H_2_O (4 × 100 mL). The combined organic layers were dried over MgSO_4_, and concentrated under reduced pressure. Purification of the crude residue by reversed-phase chromatography on Lichroprep@RP-18 was eluted with ACN gradient grade to get compound 7 (46 mg, 0.021 mmol, 30%). ^1^H NMR (400 MHz, CDCl3) δ 8.50–8.44 (m, 4H), 7.68 (d, *J* = 7.9 Hz, 2H), 7.64–7.52 (m, 10H), 7.17 (t, *J* = 8.7 Hz, 5H), 7.11 (ddd, *J* = 6.8, 4.9, 1.9 Hz, 5H), 6.95 (br, 2H), 6.87 (br, 2H), 6.77 (d, *J* = 9.5 Hz, 4H), 6.68–6.61 (m, 1H), 6.43 (dd, *J* = 15.3, 11.1 Hz, 1H), 6.26 (d, *J* = 1.2 Hz, 1H), 5.63 (dd, *J* = 15.3, 9.1 Hz, 1H), 5.43 (t, *J* = 6.9 Hz, 1H), 4.72 (dd, *J* = 12.1, 3.1 Hz, 1H), 4.54 (s, 1H), 4.27 (ddd, *J* = 12.5, 10.5, 2.0 Hz, 2H), 4.09 (s, 1H), 3.93 (s, 4H), 3.89 (s, 2H), 3.78 (s, 10H), 3.59 (t, *J* = 15.3 Hz, 14H), 3.48 (d, *J* = 9.0 Hz, 2H), 3.38–3.28 (m, 5H), 3.23 (dt, *J* = 13.5, 6.7 Hz, 3H), 3.07 (d, *J* = 17.6 Hz, 5H), 3.00 (d, *J* = 9.6 Hz, 2H), 2.88 (d, *J* = 8.3 Hz, 1H), 2.79 (s, 4H), 2.72–2.60 (m, 1H), 2.56 (dd, *J* = 14.4, 12.1 Hz, 1H), 2.29 (s, 6H), 2.24–2.09 (m, 6H), 1.99 (q, *J* = 6.5 Hz, 1H), 1.56–1.49 (m, 2H), 1.49–1.37 (m, 2H), 1.34–1.16 (m, 18H), 0.90–0.82 (m, 1H), 0.78 (s, 3H). ^13^C NMR (151 MHz, CDCl_3_) δ 170.81, 170.76, 168.83, 159.81, 155.85, 152.32, 149.03, 144.52, 142.05, 141.04, 140.68, 139.55, 136.55, 133.42, 132.44, 130.01, 129.14, 127.65, 127.35, 126.88, 126.42, 125.34, 122.85, 122.08, 118.69, 113.58, 113.19, 88.68, 80.90, 78.33, 74.21, 70.42, 70.03, 68.73, 67.43, 60.10, 59.98, 58.68, 56.69, 56.63, 53.54, 52.40, 51.11, 46.65, 39.84, 38.94, 36.96, 36.35, 36.01, 35.30, 34.15, 32.44, 31.99, 30.55, 29.86, 29.78, 29.70, 29.64, 29.60, 29.57, 29.44, 29.40, 29.34, 27.30, 25.93, 25.89, 25.65, 24.27, 23.75, 22.77, 15.64, 14.66, 14.21, 13.45, 12.10. HRMS(ESI) calculated for C_119_H_139_ Cl_3_ N_12_NaO_17_S^+^: 2167.9060, found: 2167.9042 (M + Na^+^)^+^.

### 2.7. (14S,16S,32R,33S,2R,4S,10E,12E,14R)-86-Chloro-14-hydroxy-85,14-dimethoxy-33,2,7,10-tetramethyl-12,6-dioxo-7-aza-1(6,4)-oxazinana-3(2,3)-oxirana-8(1,3)-benzenacyclotetradecaphane-10,12-dien-4-yl N-(3-((4-(12-(4-((N-(4-(3,5-bis((bis(pyridin-2-ylmethyl)amino)methyl)phenoxy)butyl)-1-(4-chlorophenyl)cyclohexane-1-carboxamido)methyl)phenyl)-2-(1-(4-chlorophenyl)cyclohexane-1-carbonyl)-12-oxo-5,8-dioxa-2,11-diazadodecyl)benzoyl)thio)propanoyl)-N-methyl-D-alaninate*2[Zn(NO_3_)_2_]: Zn8_DM1

To a stirred solution of compound 7 (46 mg, 0.021 mmol) in 2 mL EtOH, 1 mL of Zn(NO_3_)_2_ (12.4 mg, 0.042 mmol) in EtOH at room temperature was added. After 10 min, the mixture was concentrated under reduced pressure, and the products were precipitated in ether to recover 50 mg of white solid (**Zn8_DM1**, 0.019 mmol, 90%). ^1^H NMR (600 MHz, D_6_-DMSO) δ 8.73–8.63 (m, 3H), 8.45 (s, 1H), 8.15–8.05 (m, 3H), 7.78 (s, 2H), 7.69–7.61 (m, 3H), 7.56 (s, 3H), 7.49 (s, 1H), 7.39 (d, *J* = 8.3 Hz, 5H), 7.23 (s, 5H), 7.11 (s, 1H), 7.08–6.97 (m, 2H), 6.91 (s, 3H), 6.66 (d, *J* = 11.5 Hz, 1H), 6.59 (d, *J* = 14.7 Hz, 1H), 6.56 (d, *J* = 1.8 Hz, 1H), 5.93 (d, *J* = 1.5 Hz, 1H), 5.49 (t, *J* = 12.2 Hz, 1H), 5.35 (q, *J* = 6.9 Hz, 1H), 4.55 (s, 2H), 4.46 (d, *J* = 12.1 Hz, 2H), 4.42–4.30 (m, 4H), 4.30–4.11 (m, 3H), 4.11–4.02 (m, 2H), 3.96–3.70 (m, 10H), 3.64 (d, *J* = 12.4 Hz, 1H), 3.58–3.40 (m, 8H), 3.25 (s, 7H), 3.16–3.03 (m, 2H), 3.03–2.82 (m, 5H), 2.78 (d, *J* = 9.6 Hz, 1H), 2.68 (s, 3H), 2.56 (s, 1H), 2.45 (t, *J* = 13.1 Hz, 1H), 2.27 (s, 2H), 2.13 (s, 2H), 2.04–1.93 (m, 2H), 1.77–1.47 (m, 17H), 1.47–1.38 (m, 2H), 1.31–1.14 (m, 11H), 1.11 (d, *J* = 6.2 Hz, 3H), 1.08–1.00 (m, 2H), 0.84 (t, *J* = 6.9 Hz, 1H), 0.75 (s, 3H). ^13^C NMR (151 MHz, D_6_-DMSO) δ 174.32, 173.76, 170.37, 168.12, 155.02, 154.32, 151.25, 147.93, 144.68, 144.46, 141.05, 140.92, 140.77, 138.90, 133.71, 132.80, 131.05, 129.66, 128.81, 128.12, 127.21, 126.49, 126.15, 124.89, 124.62, 117.63, 116.94, 113.80, 88.25, 80.01, 77.72, 73.21, 69.72, 69.53, 68.93, 67.73, 66.95, 59.97, 56.92, 56.47, 56.15, 56.05, 55.70, 51.60, 50.50, 45.43, 37.78, 36.31, 35.13, 34.60, 33.39, 31.86, 31.29, 29.50, 29.09, 29.03, 28.87, 28.84, 28.75, 28.70, 28.59, 26.57, 25.28, 25.13, 23.73, 23.21, 22.10, 18.57, 15.15, 14.45, 13.96, 13.04, 11.20. HRMS(ESI) calculated for C_119_H_139_Cl_3_ N_12_O_17_SZn^2+^: 1104.9241, found: 1104.9232(M + Zn^2+^)^2+^.

### 2.8. (14R,16R,32S,33R,2S,4R,10E,12E,14S)-86-Chloro-14-hydroxy-85,14-dimethoxy-33,2,7,10-tetramethyl-12,6-dioxo-7-aza-1(6,4)-oxazinana-3(2,3)-oxirana-8(1,3)-benzenacyclotetradecaphane-10,12-dien-4-yl N-(4-((4-(12-(4-((N-(4-(3,5-bis((bis(pyridin-2-ylmethyl)amino)methyl)phenoxy)butyl)-1-(4-chlorophenyl)cyclohexane-1-carboxamido)methyl)phenyl)-2-(1-(4-chlorophenyl)cyclohexane-1-carbonyl)-12-oxo-5,8-dioxa-2,11-diazadodecyl)benzoyl)thio)-4-methylpentanoyl)-N-methyl-L-alaninate (***9***)

To a stirred solution of compound 6 (100 mg, 0.070 mmol) dissolved in DMF (3 mL), (14R,16R,32S,33R,2S,4R,10E,12E,14S)-86-chloro-14-hydroxy-85,14-dimethoxy-33,2,7,10-tetramethyl-12,6-dioxo-7-aza-1(6,4)-oxazinana-3(2,3)-oxirana-8(1,3)-benzenacyclotetradecaphane-10,12-dien-4-yl *N*-(4-mercapto-4-methylpentanoyl)-N-methyl-L-alaninate (DM-4) (70 mg, 0.089 mmol, 1.2 equiv.), 4-dimethylaminopyridine (DMAP, 15 mg, 0.122 mmol, 1.7 equiv.), and *N*-(3-dimethylaminopropyl)-*N*′-ethylcarbodiimide hydrochloride (EDCl, 15 mg, 0.096 mmol, 1.3 equiv.) were added. The mixture was stirred at room temperature in the dark for 15 h. After completion of the reaction, the mixture was extracted with CH_2_Cl_2_ (100 mL), saturated aqueous NaHCO_3_ (100 mL), and H_2_O (4 × 100 mL). The combined organic layers were dried over MgSO_4_, and concentrated under reduced pressure. Purification of the crude residue by reversed-phase chromatography on Lichroprep@RP-18 was eluted with ACN gradient grade to get compound 9 (20 mg, 0.009 mmol, 12%). ^1^H NMR (400 MHz, CDCl3) δ 8.54–8.43 (m, 4H), 7.70 (d, *J* = 7.8 Hz, 2H), 7.65–7.54 (m, 8H), 7.51 (d, *J* = 8.1 Hz, 2H), 7.29 (d, *J* = 1.9 Hz, 1H), 7.27 (d, *J* = 2.1 Hz, 1H), 7.24 (s, 1H), 7.20 (d, *J* = 2.0 Hz, 2H), 7.18 (d, *J* = 2.1 Hz, 2H), 7.16 (s, 1H), 7.13 (d, *J* = 1.8 Hz, 1H), 7.12 (t, *J* = 1.9 Hz, 2H), 7.11 (d, *J* = 1.9 Hz, 1H), 6.96 (br, 2H), 6.89 (br, 2H), 6.78 (dd, *J* = 11.4, 7.6 Hz, 4H), 6.53 (d, *J* = 1.8 Hz, 1H), 6.41 (dd, *J* = 15.3, 11.1 Hz, 1H), 6.26 (s, 1H), 5.69 (dd, *J* = 15.3, 9.0 Hz, 1H), 5.41 (q, *J* = 6.8 Hz, 1H), 5.33 (td, *J* = 4.5, 2.2 Hz, 1H), 4.74 (dd, *J* = 12.1, 3.0 Hz, 1H), 4.55 (s, 1H), 4.31–4.15 (m, 2H), 4.10 (s, 1H), 3.94 (s, 2H), 3.78 (s, 8H), 3.71–3.51 (m, 16H), 3.48 (d, *J* = 9.0 Hz, 2H), 3.32 (s, 3H), 3.25 (d, *J* = 6.7 Hz, 2H), 3.18–3.08 (m, 2H), 3.01 (d, *J* = 9.7 Hz, 2H), 2.95 (s, 2H), 2.79 (s, 2H), 2.59–2.43 (m, 2H), 2.43–2.30 (m, 3H), 2.26–2.14 (m, 5H), 2.10 (dd, *J* = 14.3, 3.0 Hz, 1H), 2.05–1.96 (m, 2H), 1.94 (s, 7H), 1.52 (s, 4H), 1.42 (d, *J* = 9.2 Hz, 4H), 1.36–1.18 (m, 20H), 1.16 (t, *J* = 7.3 Hz, 1H), 1.09 (t, *J* = 6.9 Hz, 1H), 0.91–0.81 (m, 2H), 0.76 (s, 3H). ^13^C NMR (151 MHz, CDCl_3_) δ 172.19, 171.30, 169.03, 160.07, 156.06, 152.56, 149.28, 142.22, 141.73, 140.93, 139.36, 136.78, 133.65, 132.69, 130.25, 129.40, 128.04, 127.30, 127.07, 126.63, 125.78, 123.09, 122.43, 122.31, 118.79, 113.82, 113.41, 88.86, 81.18, 78.38, 74.40, 70.67, 70.31, 67.57, 60.36, 60.25, 58.93, 56.92, 56.78, 52.62, 51.37, 47.02, 45.72, 40.10, 39.11, 37.20, 36.55, 36.26, 35.38, 32.60, 32.24, 30.92, 30.29, 30.11, 30.03, 29.94, 29.82, 29.65, 29.58, 29.05, 28.30, 27.55, 26.15, 25.88, 24.00, 23.02, 15.78, 14.87, 14.45, 13.58, 12.39. HRMS(ESI) calculated for C_122_H_145_ Cl_3_ N_12_NaO_17_S^+^: 2209.9529, found: 2209.9518 (M + Na^+^)^+^.

### 2.9. (14R,16R,32S,33R,2S,4R,10E,12E,14S)-86-Chloro-14-hydroxy-85,14-dimethoxy-33,2,7,10-tetramethyl-12,6-dioxo-7-aza-1(6,4)-oxazinana-3(2,3)-oxirana-8(1,3)-benzenacyclotetradecaphane-10,12-dien-4-yl N-(4-((4-(12-(4-((N-(4-(3,5-bis((bis(pyridin-2-ylmethyl)amino)methyl)phenoxy)butyl)-1-(4-chlorophenyl)cyclohexane-1-carboxamido)methyl)phenyl)-2-(1-(4-chlorophenyl)cyclohexane-1-carbonyl)-12-oxo-5,8-dioxa-2,11-diazadodecyl)benzoyl)thio)-4-methylpentanoyl)-N-methyl-L-alaninate*2[Zn(NO_3_)_2_]: Zn10_DM4

To a stirred solution of compound 9 (20 mg, 0.009 mmol) in 2 mL, 1 mL of Zn(NO_3_)_2_ (5.3 mg, 0.018 mmol) in EtOH at room temperature was added. After 10 min, the mixture was concentrated under reduced pressure and products were precipitated in ether to recover 23 mg of white solid (**Zn10_DM4**, 0.008 mmol, 90%). ^1^H NMR (600 MHz, D_6_-DMSO) δ 8.68 (d, *J* = 5.3 Hz, 3H), 8.45 (s, 1H), 8.19–7.99 (m, 3H), 7.78 (s, 2H), 7.65 (t, *J* = 6.4 Hz, 3H), 7.56 (s, 3H), 7.39 (t, *J* = 10.0 Hz, 5H), 7.32–7.14 (m, 6H), 7.07 (s, 1H), 7.02 (d, *J* = 17.6 Hz, 2H), 6.96–6.89 (m, 2H), 6.87 (s, 1H), 6.66–6.58 (m, 1H), 6.53 (dd, *J* = 15.0, 11.2 Hz, 1H), 6.39 (s, 1H), 5.91 (s, 1H), 5.61 (dd, *J* = 15.0, 9.1 Hz, 1H), 5.40–5.23 (m, 2H), 4.63–4.41 (m, 3H), 4.41–4.30 (m, 3H), 4.17 (s, 2H), 4.11–3.96 (m, 2H), 3.86 (s, 6H), 3.78 (d, *J* = 15.8 Hz, 4H), 3.63 (s, 1H), 3.57–3.41 (m, 8H), 3.25 (s, 6H), 3.09 (s, 1H), 3.04–2.83 (m, 4H), 2.77 (d, *J* = 9.6 Hz, 1H), 2.67 (s, 2H), 2.57 (d, *J* = 14.9 Hz, 1H), 2.41 (t, *J* = 13.0 Hz, 1H), 2.29 (d, *J* = 15.6 Hz, 4H), 2.13 (s, 3H), 2.05–1.90 (m, 4H), 1.78–1.48 (m, 20H), 1.44 (td, *J* = 11.9, 5.0 Hz, 3H), 1.35 (d, *J* = 17.9 Hz, 3H), 1.31–1.16 (m, 13H), 1.13 (d, *J* = 6.7 Hz, 2H), 1.10 (d, *J* = 6.4 Hz, 2H), 1.05 (t, *J* = 7.0 Hz, 1H), 0.85 (t, *J* = 7.0 Hz, 1H), 0.73 (s, 2H). ^13^C NMR (151 MHz, D_6_-DMSO) δ 174.57, 173.99, 171.41, 171.10, 168.36, 155.33, 154.58, 151.53, 148.19, 144.78, 141.16, 141.03, 138.45, 133.98, 132.95, 131.34, 129.93, 129.10, 128.81, 127.45, 126.57, 125.47, 125.16, 124.88, 121.62, 117.91, 117.21, 114.02, 88.50, 80.28, 77.82, 73.42, 69.97, 69.80, 69.21, 67.06, 60.27, 57.21, 56.65, 56.39, 56.31, 55.98, 51.97, 51.18, 50.78, 45.93, 40.33, 37.88, 36.62, 35.92, 35.40, 34.83, 32.04, 31.56, 30.64, 29.94, 29.50, 29.36, 29.30, 29.25, 29.14, 29.11, 29.02, 28.97, 28.85, 27.30, 26.83, 25.54, 25.39, 23.49, 22.37, 18.84, 15.25, 14.67, 14.22, 13.30, 11.63. HRMS(ESI) calculated for C_122_H_145_Cl_3_N_12_O_17_SZn^2+^: 1125.9475, found 1125.9469 (M + Zn^2+^)^2+^.

### 2.10. Cell Culture/Viability Assay/Data Analysis

HCC1806 cells were grown in RPMI 1640 medium, and MTS assays were performed to check cell viability. Cells (2500–3000 cells/well) were grown in flat-bottomed 96-well plates for 24 h, and then serial dilutions of the compound were added, and cells were allowed to incubate for a further 72 h. At the end of the 72-h incubation period, the medium was removed, and 100 μL of a solution including a mixture of MTS and PMS was added. The cells were incubated for 1.5 h at 37 °C in a humidified incubator with 5% CO_2_ to allow viable cells to convert tetrazolium salts to formazan. Conversion to formazan was measured by absorbance (490 nm) using a BioTek PowerWave-X absorbance microplate meter. The data collected were normalized using a DMSO-treated control (100% viability) and a background control (0% viability) to verify growth inhibition. At the same time, IC_50_ values were calculated using GraphPad Prism version 4 software (San Diego, CA, USA), i.e., the reduction in cell viability compared to the DMSO-treated control resulted in the amount of compound that resulted in a 50% reduction in cell viability compared to the DMSO-treated control.

### 2.11. PS SPR Binding Assay

Phospholipids, 1,2-dioleoyl-sn-glycero-3-phosphocholine (DOPC) and 1,2-dioleoyl-sn-glycero-3-[phosphate-L-serine] (DOPS) were obtained as chloroform solutions from Avanti Polar Lipids (Alabaster, AL, USA). These stock solutions were mixed in the specified proportions. A 0.4 mL aliquot of the lipid solution at a 10 mg/mL concentration was added to a round bottom flask and evaporated under a stream of N_2_ to provide a thin lipid film. The lipid films were rehydrated in PBS buffer for at least 1 h at room temperature. The resulting suspension was extruded through a 100 nm polycarbonate filter using an Avanti MiniExtruder according to the manufacturer’s instructions. Zeta potential (ZP) and liposome size distribution were recorded by dynamic light scattering (DLS) and microelectrophoresis using a Zetasizer Nano ZS instrument. The binding kinetics between conjugates and liposomes were recorded at 25 °C using a Biacore T200 biosensor equipped with an L1 sensor chip (GE Healthcare, Chicago, IL, USA). The new sensor chip was pretreated with running buffer (5% DMSO in phosphate buffered saline (PBS) at a pH of 7.4) and two consecutive 30 s pulses of 2:3 *v*/*v* 50 mM HCl/isopropanol at a flow rate of 30 μL/min. New liposome capture plates were prepared for each binding cycle. In PBS buffer, liposomes are diluted to 0.5–1 mM and captured to saturation in isolated flow cells at 2–5 μL/min (30–150 s). The conjugate is first diluted with running buffer and injected onto the liposome surface in one injection. The conjugation and dissociation phases were examined for 60 s at a flow rate of 30 μL/min. At the end of each binding cycle, the surface was regenerated by injecting 2:3 *v*/*v* 50 mM hydrochloric acid/isopropanol and equilibrated with running buffer before the next injection of the test compound. Non-specific binding was removed by subtracting the SPR signal from the reference flow cell (DOPC immobilized surface). The sensory maps were fully fitted using a bivalent analyte model with BIAcore T200 evaluation software 3.0.

### 2.12. IVIS Imaging of HCC1806 Tumors

HCC1806 tumor-bearing mice were used when the average tumor volume reached approximately 500–600 mm^3^. Tumor volume (mm^3^) was calculated by the formula. Volume = (length × width^2^)/2, measured with digital calipers. The non-targeting dye 794 at a dose of 2 mg/kg was administered intravenously. All treated mice were imaged at 24 and 48 h using the IVIS spectroscopy system. Briefly, mice were anaesthetized by inhalation of 2.5% isoflurane and placed on the platform of the IVIS instrument (IVIS^®^ Spectrum, PerkinElmer, Waltham, MA, USA) with the following imaging conditions set: excitation filter, 745 nm; emission filter, 820 nm; exposure time, 1 s, bin, 8 (medium); f/stop, 2; field of view, 22.7 cm. Fluorescence intensity was quantified, and images were processed using Living Image 4.5 software (PerkinElmer, Alameda, CA, USA).

### 2.13. Pharmacokinetic Studies of Conjugates

Six-week-old male ICR mice from BioLASCO Taiwan were divided into three groups and administered intravenously at a 5 mg/kg dose. Blood samples were taken from each animal at time points 0.003, 0.083, 0.25, 0.5, 1, 2, 4, 6, 8, and 24 h and stored on ice (0–4 °C). The plasma was separated from the blood by centrifugation (in a Beckman Allegra 6R at 3000 rpm, 4 °C for 15 min) and stored under freezing conditions (−20 °C). Plasma and harvested tumor samples were stored at −80 °C until use. Fifty microliters of mouse plasma or tumor samples homogenized with ddH_2_O at a dilution ratio of 1:3 (*w*/*v*) with MiniBeadbeater-16 (BioSpec Products Inc., Bartlesville, OK, USA) were mixed with 100 μL of acetonitrile containing 250 ng/mL BPR0L187. The mixture was vortexed for 30 s and then centrifuged at 15,000× *g* for 20 min. The supernatant was transferred to a clean test tube, and 15 µL of supernatant was injected into the LC/MS/MS. Plasma samples were analyzed by liquid chromatography-tandem mass spectrometry (LC/MS/MS). The chromatography system was an Agilent 1200 series liquid chromatography system and an Agilent ZORBAX Eclipse XDB-C8 column (5 µm, 3.0 × 150 mm) connected with an MDS Sciex API4000 tandem mass spectrometer equipped with an ESI in forwarding scan mode at 600 °C. Data acquisition was performed by multiple reaction monitoring (MRM). A gradient system was used to separate the analytes from the IS. Mobile phase A was a 10 mM aqueous ammonium acetate solution containing 0.1% formic acid. Mobile phase B was acetonitrile. The gradient profile was as follows. 0.0–1.1 min, 50% B; 1.2–3.7 min, 55% B-90% B; 3.8–5.0 min, 90% B-50% B. The flow rate was 1.5 mL/min. The autosampler was set to inject 15 µL of the sample every 5 min.

## 3. Animal Studies

Cancer cells suspended in RPMI 1640 medium (11835-030, Thermo Fisher Scientific, Waltham, MA, USA) were mixed with Matrigel™ (356237, BD Biosciences, Franklin Lakes, NJ, USA) in a 1:1 ratio. Human triple-negative breast cancer HCC1806 (1 × 10^6^ cells) cells were inoculated subcutaneously into the left flank of 6-week-old female nude mice (BioLASCO Taiwan, Taipei, Taiwan) using a 1 mL syringe with a 24G needle (AN-2425R, Terumo Medical Corp., Tokyo, Japan). Tumor dimensions were measured twice weekly using electronic calipers (FOW54-200-777, PRO-MAX), and the volume of tumor grown subcutaneously (mm^3^) was calculated by the formula Volume = (length × width^2^)/2. A combination of 10% DMA/20% Cremophor EL/70% 5% dextrose injection (D5W) was formulated to treat HCC1806 xenograft tumors. Mice with HCC1806 tumors were grouped and administered the indicated dosing regimen of 1 mg/kg for the conjugate and 0.3 mg/kg for the cytotoxic payloads DM1 and DM4 twice-weekly (day 1 and day 4) for 2 weeks when the mean tumor volume was approximately 200–250 mm^3^ or 850–900 mm^3^ (large tumors). The conjugate dose of 2 mg/kg was administered weekly for the large tumor study. The body weight and tumor volume of mice were measured twice weekly. In addition, a previously reported oncogene-induced, sorafenib-resistant HCC mouse model [26] was used in the current study to examine the antitumor activities of synthesized compounds. Briefly, plasmids including transposase (pCMV(CAT)T7-SB100), human Akt1, and luciferase (pKT2/CLP-AKT-2A-OVA-HBc-HBs-LUC) and the N-Ras V12 mutant (pT/Caggs-NRASV12) were hydrodynamically injected (HDI) and the liver was monitored for the appearance of HCC. HCC mice with a total flux of more than 1 × 10^9^ photons/s from IVIS imaging were used as treatment conjugates. The indicated conjugate was administered intravenously at 1 mg/kg 3 times per week over 2 weeks. IVIS imaging was repeatedly performed to follow tumor progression, and tumor tissue from treated HCC mice was collected for histological examination and RNA extraction after administration.

### 3.1. Immunohistochemical Staining

Paraffin-embedded liver/tumor tissue sections were de-paraffined, re-hydrated, and then subjected to heat-induced antigen retrieval, followed by incubation with primary Abs. The primary antibodies used to detect Ki-67 and CD8α were SP6 (Abcam, Cambridge, UK) and D4W2Z (Cell Signaling Technology, Danvers, MA, USA), respectively. ImmPRESS anti-rat Ig, ImmPRESS anti-rabbit Ig, polymer detection kit, DAB peroxidase substrate kit (Vector laboratories, Burlingame, CA, USA), and liquid permanent red substrate (Dako, Glostrup, Denmark) and Hematoxylin Gill II (Leica, Wetzlar, Germany) were used for detection and visualization. Images were taken by NHRI’s Pathology Core Laboratory using an automated digital slide scanner Pannoramic MIDI with a Plan-Apochromat 20×/0.8 objective (3D HISTECH, Budapest, Hungary).

### 3.2. Nanostring Analysis

Total RNA was extracted from conjugate-treated or vehicle-treated HCC mouse tumor tissue using the RNA-easy kit (QIAGEN, Hilden, Germany). The concentration (absorbance at 260 nm) and purity (A260/280 and A260/230 ratios) of the extracted RNA was measured spectrophotometrically; RNA integrity was further determined using the 2100 Bioanalyzer System (Agilent RNA was further determined for RNA integrity using a 2100 Bioanalyzer System (Agilent Technologies, Santa Clara, CA, USA). RNA was hybridized to the barcode probe provided in the nCounter Mouse Pan-Cancer Immunoassay Kit (NanoString Technologies, Seattle, WA, USA) and then used to measure mRNA expression of 770 genes associated with the immune response. Nanostring nSolver 4.0 and nCounter Advanced Analysis 2.0 software (NanoString Technologies) was used for data processing, immune cell analysis, and pathway scoring according to the developer’s instructions.

### 3.3. Ethics Approval

The mice used in this study were maintained and treated according to the animal protocols (NHRI-IACUC-106076-A (approval date: 9 May 2017), NHRI-IACUC-107130-A (approval date: 2 November 2018), and NHRI-IACUC-109063 (approval date: 7 April 2020)) approved by National Health Research Institutes’ Institutional Animal Care and Use Committee (NHRI-IACUC).

### 3.4. Statistical Analysis

GraphPad Prism 7 (GraphPad Software, La Jolla, CA, USA) and Student’s *t*-test were used for statistical analysis.

## 4. Results and Discussion

The release of thioester-linked maytansinoid can be modulated by installing cyclohexyl-para-chlorophenyl steric hindered functional groups in the proximity. The longevity of the intact conjugate in systemic circulation serves as a key parameter during treatment in facilitating the selective accumulation of such a conjugate at the tumor site. As for targeting prodrugs, we envision that maytansinoid cytotoxicity is initially masked in the systemic circulation, and through the engagement of the targeting ligand, the payload was then uncaged at the tumor site. 

**Scheme 1:**
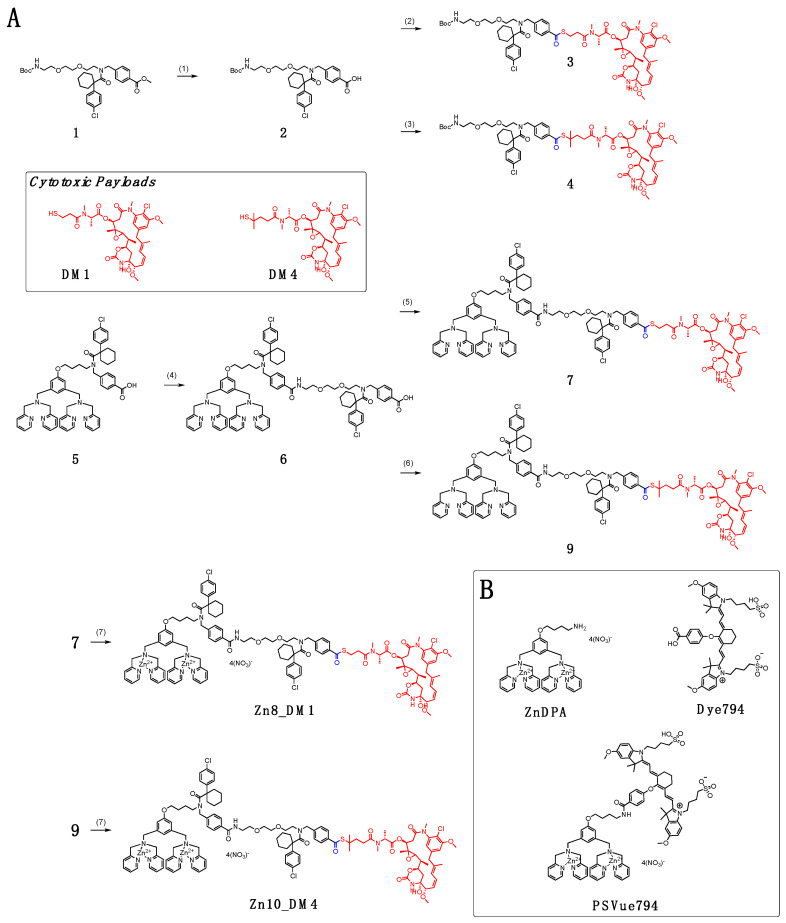
(**A**) Synthetic procedures for linker-maytansinoids **3**, **4** and their respective ZnDPA conjugates **Zn8_DM1** and **Zn10_DM4**. (**B**) Chemical structures of **ZnDPA**, **Dye_794**, and **PSVue794**. Reagents and conditions: (1) 0.5M LiOH_(aq.)_, MeOH, 15 h, 90%; (2) DM1, EDCI, DMAP, DMF, 15 h, 40%; (3) DM4, EDCI, DMAP, DMF, 15 h, 28%; (4) (a) compound 1, TFA, 3 h, 95%; (b) EDCI, HOBt, NMM, DMF, 40 °C, 15 h, 40%; (c) LiOH, 15 h, 82%; (5) DM1, EDCI, DMAP, DMF, 15 h, 30%; (6) DM4, EDCI, DMAP, DMF, 15 h, 12%; (7) 2.0 equiv. Zn(NO_3_)_2_, EtOH.

In Scheme 1A, conjugation between Boc-protected amino polyethylene glycol (PEG) with cyclohexyl-para-chlorophenyl periphery carboxylic acid **2** and maytansinoids DM1 and DM4 afforded linker payloads **3** and **4**, respectively. We installed a small-unit linear PEG to bridge the targeting ligand and the cytotoxic payload. Because large-unit pegylation can significantly increase the solubility of the conjugate, it might result in steric interference, leading to loss of the targeting ligand’s binding ability [27].

To incorporate the linker-maytansinoids into the small molecule drug conjugate (SMDC) form, we first coupled the dipicolylamine (DPA) carboxylic acid **5** with the amino PEG harnessing cyclohexyl-para-chlorophenyl periphery that yielded the carboxylic acid precursor **6**. We have shown that cyclohexyl-para-chlorophenyl in **6** resulted in an advantageous payload release profile and reduced toxicity of the released ZnDPA ligand [18]. With the activation of the carboxylic acid **6** by EDCl and HOBt in dry *N*, *N*-dimethylformamide, synthesis of new DPA-maytansinoid conjugates with pegylated linkers was then carried out with coupling of DM1 or DM4 to furnish conjugate **7** and **9**, respectively. Incubating each DPA–drug conjugate **7** or **9** with two equivalents of zinc nitrate at room temperature in 1:1 dichloromethane/methanol provided the eventual ZnDPA conjugates, **Zn8_DM1** and **Zn10_DM4**.

**Figure 1:**
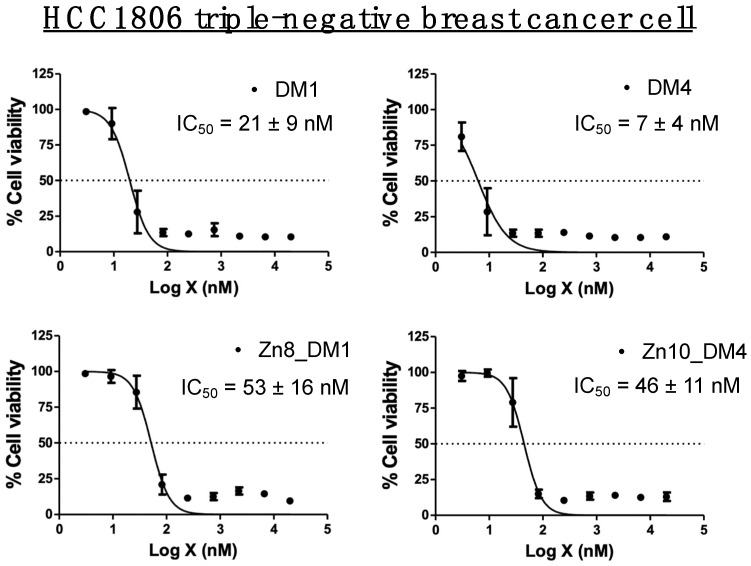
Cytotoxic effects on human triple-negative breast cancer HCC1806 cells. With a 72 h incubation of conjugates **Zn8_DM1**, **Zn10_DM4**, cytotoxic maytansinoids **DM1**, and **DM4** with the cells, reduction of tetrazolium by viable cells was determined.

We first examined plasma stabilities and in vitro cytotoxicities of maytansinoids and their respective conjugates **Zn8_DM1** and **Zn10_DM4** against the HCC1806 triple-negative breast cancer cell line (Figure 1). No cleavage of the maytansinoids from either the linker-drug **3** and **4** or conjugates **Zn8_DM1** and **Zn10_DM4** were observed after 24 h incubation with plasma (Supporting Figure S1). The cytotoxic payloads DM1 and DM4 inhibited HCC1806 at 21 nM and 7 nM, respectively. By this targeting prodrug strategy, new conjugates **Zn8_DM1** (53 nM) and **Zn10_DM4** (46 nM) exhibited 2.5–6 fold attenuation in cytotoxicities against HCC1806 triple-negative breast cancer cells relative to their parent payloads, suggesting that the thioester-linked maytansinoids shielded their cytotoxic properties in vitro (Figure 1).

**Figure 2:**
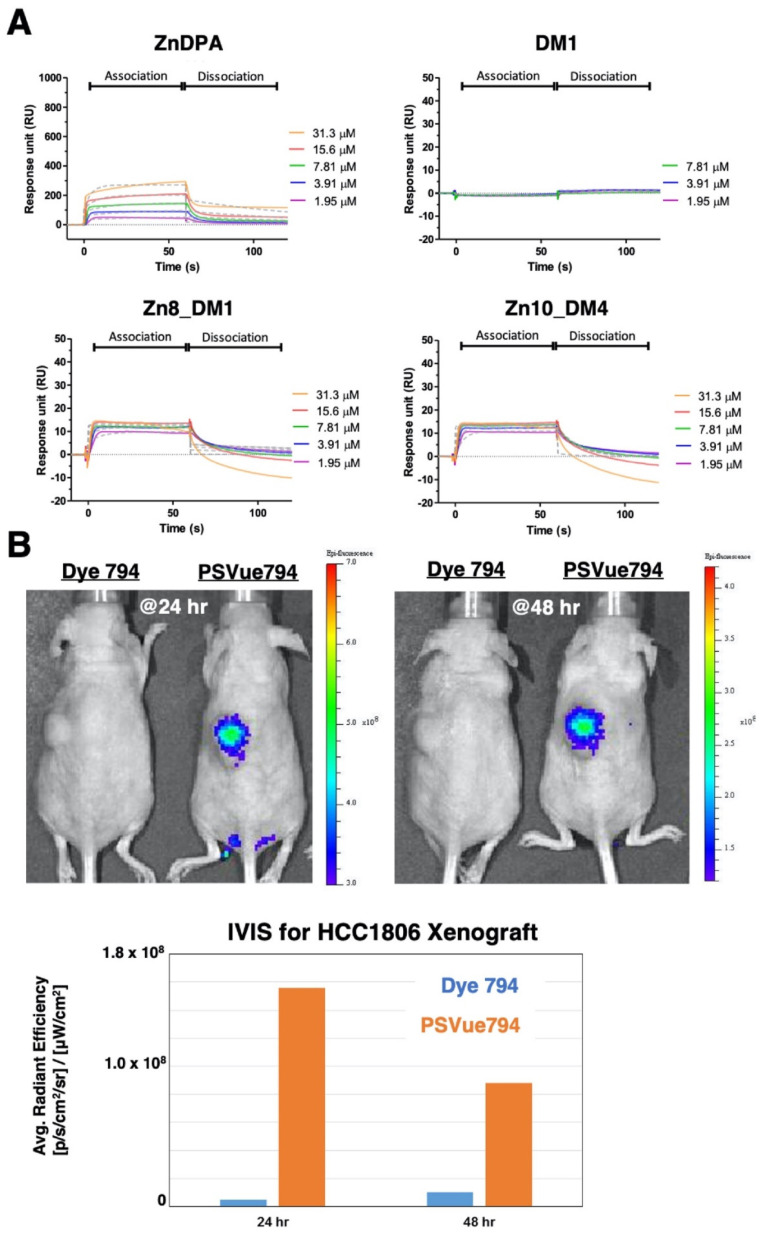
(**A**) In vitro SPR PS-association studies. Sensorgrams are generated using a Biacore T200. **ZnDPA**, **Zn8_DM1**, **Zn10_DM4**, and **DM1** were analyzed across a two-fold serial concentration dilution. The association of liposome (DOPC/ DOPS (3:1, *v*/*v*)) immobilized on an L1 chip at 5000 RU was monitored for 1 min and the dissociation time was 1 min. (**B**) In vivo targeting ability of ZnDPA and detection of PS-expression in the HCC1806 tumor xenograft model. Representative IVIS images of fluorescence probe PSVue794 and Dye_794 in mice at the indicated time-point with a single intravenous dose of 2 mg/kg.

To probe the utility of such thioester-linked maytansinoids in SMDC, we first assessed whether this addition of linker-drug allowed the ZnDPA motif to retain its’ binding property towards phosphatidylserine (PS). As the attachment of a therapeutic payload near the small molecule targeting ligand might diminish receptor binding, we performed in vitro binding studies through surface plasmon resonance (SPR) measurements between **Zn8_DM1** or **Zn10_DM4** and PS-coated liposomes (DOPC/ DOPS (3:1, *v*/*v*)) according to procedures reported in the literature [28]. The maytansinoid DM1 did not show association properties with the PS-coated liposomes (Figure 2A). The ZnDPA motif played an essential role for PS recognition. We showed that the employment of linker-maytansinoids in **Zn8_DM1** or **Zn10_DM4** still allowed recognition between the ZnDPA moiety and PS-coated liposomes (Figure 2A). To examine PS expression in the HCC1806 xenograft model, a near infra-red fluorophore-conjugated ZnDPA (PSVue^®^794) was intravenously injected in vivo in comparison to the fluorophore (Dye 794) lacking the targeting moiety. We observed an efficient and lasting accumulation of PS-seeking ZnDPA probe over dye_794 at the tumor site (Figure 2B), supporting the expression of PS at the HCC1806 tumor and providing important insight into the ZnDPA-based therapeutic delivery strategy.

Leveraging the favorable plasma stabilities of the linker maytansinoids, we then evaluated the in vivo systemic stability of **Zn8_DM1** and **Zn10_DM4**. With a single intravenous dose of 5 mg/kg in the ICR mice, we profiled the in vivo systemic pharmacokinetic properties of **Zn8_DM1** and **Zn10_DM4** (Table 1). In general, small volume distributions V_ss_ (0.2~0.6 L/kg) of the maytansinoid conjugates were observed, suggesting that systemic distributions of **Zn8_DM1** and **Zn10_DM4** were limited to the circulation. With a slow and similar clearance (CL) rate at 0.5 mL/min/kg among the conjugates, **Zn8_DM1** and **Zn10_DM4** exhibited a large AUC ratio between intact conjugates and released payloads within 24 h in circulation. This data alleviated the concerns on the premature release of the respective cytotoxic payload from **Zn8_DM1** or **Zn10_DM4** and should facilitate adequate delivery of the cytotoxic cargoes at the tumor site. In particular, **Zn10_DM4** exhibited a higher intact conjugate AUC over that of **Zn8_DM1**, which might be attributed to the steric hindering with methyl groups on the adjacent carbon next to the sulfur group in DM4. By installing newly constructed thioester-linked maytansinoids in the ZnDPA-based conjugates, we demonstrated stable systemic exposure of **Zn8_DM1** or **Zn10_DM4** in vivo with PS-targeting ability and prodrug properties.

**Figure 3:**
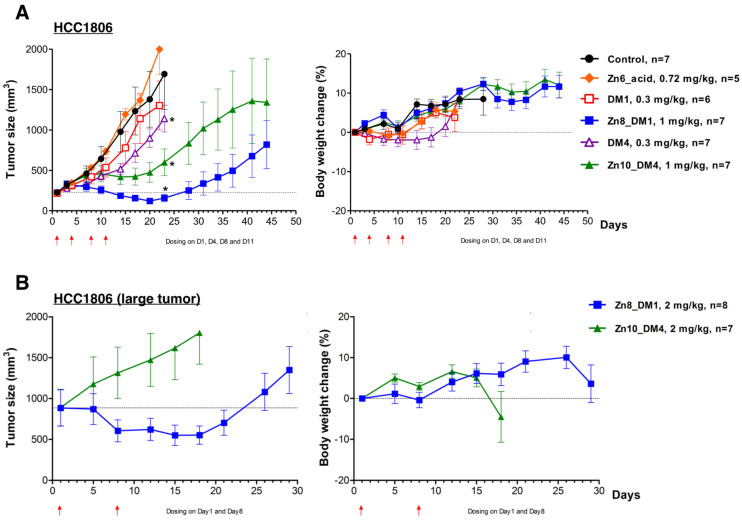
In vivo anti-HCC1806 tumor efficacies. The treatment regimen is shown in mg/kg, and dosing frequency per week is indicated by red arrows. The amount of cytotoxic maytansinoids **DM1** and **DM4** deployed was calculated from the percentage of **DM1** and **DM4** in the total dose of conjugates **Zn8_DM1** and **Zn10_DM4** used. (**A**) Comparisons of anti-HCC1806 triple-negative breast cancer activities and body weight changes between **Zn8_DM1**, **Zn10_DM4**, cytotoxic maytansinoids **DM1** and **DM4** when administered intravenously at indicated time-point. (**B**) Treatment of large (~900 mm^3^) HCC1806 triple-negative breast cancer tumor and body weight changes with weekly doses of conjugates **Zn8_DM1** and **Zn10_DM4** at 2 mg/kg, * *p*  <  0.05 via unpaired *t*-test.

ZnDPA-based thioester-linked maytansinoids **Zn8_DM1** or **Zn10_DM4** exhibited significant activities against HCC1806 human triple-negative breast cancer growth compared to vehicle control (Figure 3A). With equimolar doses of cytotoxic payloads (0.3 mg/kg for DM1 or DM4 and 1 mg/kg for **Zn8_DM1** or **Zn10_DM4**), we observed that these maytansinoid conjugates exhibited better efficacies than their respective payloads alone (Figure 3A). On the other hand, **Zn6_acid**, the part of ZnDPA-linker without the bioactive maytansinoid payloads, was intravenously given in 4 doses at 0.72 mg/kg, and did not reduce the tumor burden in mice (Figure 3A). Notably, a week after the stoppage of treatment, **Zn8_DM1** showed shrinkage of HCC1806 tumor growth and had more significant antitumor activity when compared to **Zn10_DM4**, which had a tumor growth inhibition (TGI) of 74% only. Although 0.3 mg/kg of DM4 elicited marginal improvement in its antitumor activity during the course of treatment when compared to that of DM1, in its respective conjugated form, **Zn8_DM1** showed better efficacy than that of **Zn10_DM4**. We reasoned that the introduction of methyl groups to the adjacent carbon next to the sulfur group in DM4 might generate more steric hindrance for efficient DM4 cleavage from **Zn10_DM4** in the tumor microenvironment. With a low-frequency regimen at 2 mg/kg per week for two weeks, a therapy experiment was conducted against larger (~900 mm^3^) HCC1806 tumors. Gratifyingly, our data showed that **Zn8_DM1** could readily arrest or shrink the growth of larger tumors (Figure 3B), providing insight into reducing tumor burden and facilitating surgical removal procedures for the treatment of TNBC cancer.

**Figure 4:**
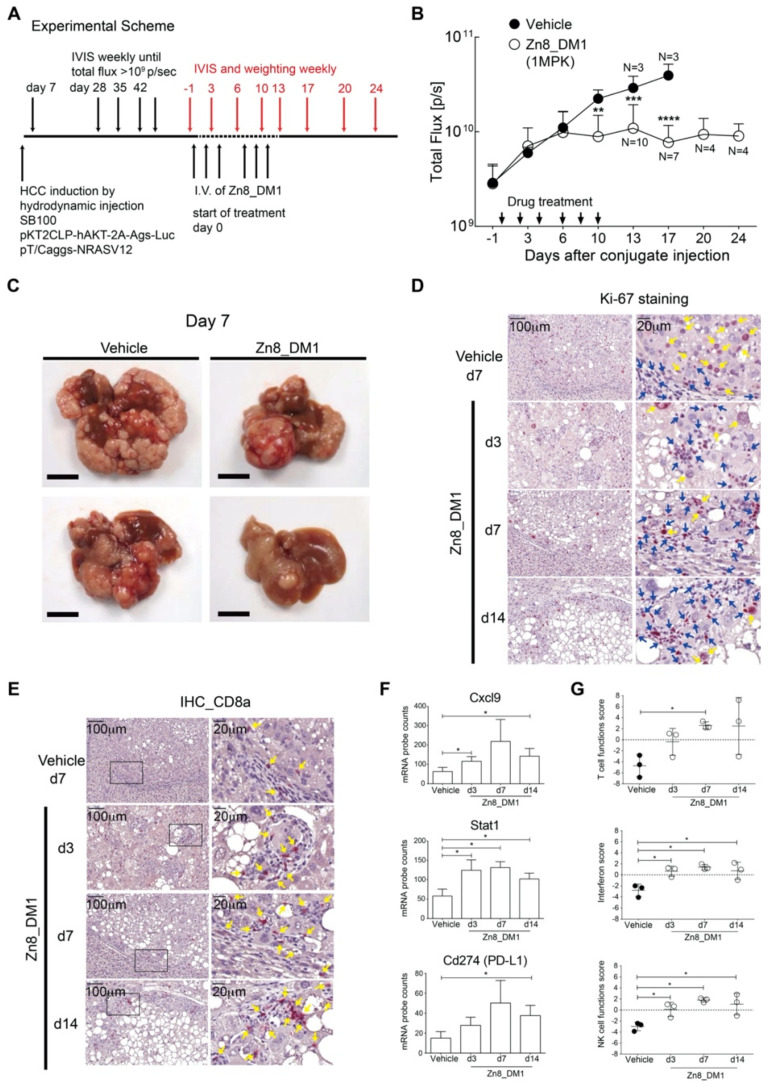
In vivo anti-liver cancer efficacies of conjugate **Zn8_DM1**. (**A**) Experimental scheme for HCC induction and treatment regimen. (**B**) Bioluminescence detection between mice treated with vehicle and conjugate **Zn8_DM1** via intravenous administration at 1 mg/kg with the indicated regimen. (**C**) Representative images of liver of vehicle- or conjugate **Zn8_DM1**-treated mice livers harvested at 7 days post drug administration. (**D**) Ki-67 staining of vehicle- or conjugate **Zn8_DM1**-treated tumor tissues at the indicated time-point. A significant reduction of Ki-67 positive cancer cells staining (yellow arrows) and increased mononuclear immune cells (blue arrows) in the **Zn8_DM1**-treated tumor was observed. Scale of 100 µm or 20 µm. (**E**) Immunohistochemical analysis (yellow arrows) of CD8α-positive cells in vehicle- or conjugate **Zn8_DM1**-treated tissues. (**F**) Quantitative gene analysis of conjugate **Zn8_DM1-**induced immunogenic gene expression in the TME. (**G**) Elevation of T cell-, NK cell-, and interferon-function in the TME in **Zn8_DM1**-treated tissue, * *p* < 0.05; ** *p* < 0.01; *** *p* < 0.001; **** *p* < 0.0001 via unpaired *t*-test.

In an oncogene-induced, sorafenib-resistant hepatocellular carcinoma mouse model with relevant tumor-associated immunological profiles in the TME, we then evaluated the efficacies of **Zn8_DM1** by non-invasive monitoring of tumor progression with a bioluminescence reporter (Figure 4A) [26]. Notably, **Zn8_DM1** achieved a potent anti-HCC tumor growth evident by reducing the flux of luciferase activity at 3 times of 1 mg/kg a week for 2 weeks (Figure 4B). Indeed, livers harvested from **Zn8_DM1**-treated mice a week post last treatment dose exhibited less tumor burden than those of the vehicle-treated group (Figure 4C). Next, a marked reduction in Ki-67 staining was found with **Zn8_DM1**-treated tumor samples (Figure 4D), suggesting **Zn8_DM1** treatment could modulate Ki-67-positive cancer cell proliferation. Moreover, inflamed “hot” tumor potentiated by T-cell infiltration with immune activation properties has been shown to improve clinical response rates to (PD-L)1/PD-1 immunotherapy treatment [29,30,31]. A significant increase in cytotoxic CD8^+^ T-cell infiltration was found in **Zn8_DM1**-treated tumors (Figure 4E). These cytotoxic T cells harnessed their killing ability via programming tumor cells to undergo apoptosis. 

We next evaluated **Zn8_DM1**-mediated changes in gene expression in the TME. To address the influence of **Zn8_DM1** on the immune landscape of HCC TME, total RNA from **Zn8_DM1**- or vehicle-treated tumor tissues was isolated to determine the absolute copies of inflammation-related mRNAs. We found a significant upregulation of CXCL9 expression in the **Zn8_DM1**-treated tumor tissues (Figure 4F). Chemokine (C-X-C motif) ligand 9 (CXCL9) was identified as a chemoattractant for CD8^+^ T cells through the interaction with C-X-C motif chemokine receptor 3 (CXCR3). The CXCL9–CXCR3 axis was correlated with improved PD-1 immunotherapy outcome [32]. On the other hand, gene expression of the signal transducer and activator of transcription 1 (STAT1), a critical inflammatory regulator of TME that potentiates the immune checkpoint blockade (ICB) treatment [29], was elevated (Figure 4F). Findings of STAT1-deficient T cells possessed less serine esterase activity with impaired production of IFNγ [33], suggesting its role in regulating cytotoxic T cell function. Furthermore, ICB-responsive inflammatory TME with elevated PD-L1 gene expression was also observed in **Zn8_DM1**-treated tumor (Figure 4F). Our results provided evidence that **Zn8_DM1** treatment could result in the concurrent increase of gene expression of key factors that aided the establishment of an immune-inflamed “hot” tumor [30]. With significant improvement in T-cell function score, NK cell function score, and interferon score, **Zn8_DM1**-treatment facilitated intrinsic functional enhancement among these parameters in the TME (Figure 4G). 

## 5. Conclusions

To the best of our knowledge, this is the first chemically distinct thioester-linked maytansinoid investigated in the form of an SMDC. We integrated the new linker maytansinoid moiety with ZnDPA to demonstrate its utility as a cancer therapeutic. By leveraging the good plasma stability of sterically hindered thioester maytansinoid and the favorable pharmacokinetic profiles in the systemic circulation of its ZnDPA conjugated form, delivery of the cytotoxic maytansinoid should be readily liberated by higher protease activities that are released from the dead cells in the acidic tumor microenvironment. Indeed, the ZnDPA-based thioester-linked maytansinoid conjugate exerted potent activities against triple-negative breast tumor, and a marked increase in CD8^+^ T cell infiltration and elevation of key gene expressions to induce a “hot inflamed” tumor were observed. The current study thus established the usage of the designed thioester-linked maytansinoid in ligand-targeting drug conjugates. The balance between the complementarity of a targeting moiety, longevity of the conjugate with stable linkers, and the drug pharmacology is essential for developing effective ligand-targeted cancer therapeutics. We anticipate that this work should motivate the construction of new therapeutic conjugates harnessing the described thioester-linked maytansinoid conjugated with a wide array of targeting ligands for cancer treatments.

## Figures and Tables

**Table 1:** In vivo pharmacokinetic profiles of conjugates **Zn8_DM1** and **Zn10_DM4** in male ICR mice (n = 3) at 5 mg/kg with intravenous administration.

Compound	CL (mL/min/kg)	Vss (L/kg)	AUC (0–24 h)	(ng/mL·h)
**Zn8_DM1**	0.5	0.6	Intact Conjugate: 161,538	DM1: 500
**Zn10_DM4**	0.5	0.3	Intact Conjugate: 182,259	DM4: 751

## Supplementary Materials

The following supporting information can be downloaded at: https://www.mdpi.com/article/10.3390/pharmaceutics14071316/s1, Figure S1: Incubation of linker-drug **3** and **4**, conjugates **Zn8_DM1** and **Zn10_DM4**, **DM1** and **DM4** with plasma for 24 h. Table S1: Chromatographic conditions and parameters.

## Abbreviations

ZnDPA, zinc dipicolylamine; ADC, antibody-drug conjugates; SMDC, small molecule drug conjugates; PS, phosphatidylserine; TME, tumor microenvironment; MMAE, monomethyl auristatin E; SPR, surface plasmon resonance.
