# Supplementary material for: Synthesis and Evaluation of Small Molecule Drug Conjugates Harnessing Thioester-Linked Maytansinoids
Source: Pharmaceutics. 2022 Jun 21;14(7):1316. doi: 10.3390/pharmaceutics14071316 (PMC9323955; doi:10.3390/pharmaceutics14071316)

Supporting Information

Synthesis and Evaluation of Small Molecule Drug Conjugates Harnessing Thioester-linked Maytansinoids

Chen-Fu Lo[1#], Tai-Yu Chiu[1#], Yu-Tzu Liu[2#], Li-Rung Huang[2#], Teng-Kuang Yeh[1 #], Kuan-Hsun Huang[1], Kuan-Liang Liu [1], Chia-Yu Hsu[1], Ming-Yu Fang[1], Yu-Chen Huang[1], Tsu-An Hsu[1], Chiung-Tong Chen[1], Lun Kelvin Tsou*[1]

[1] Institute of Biotechnology and Pharmaceutical Research, National Health Research Institutes, Miaoli 35053, Taiwan, ROC

[2] Institute of Molecular and Genomic Medicine, National Health Research Institutes, Miaoli 35053, Taiwan, ROC

Correspondence and requests for materials should be addressed to L.K.T. (kelvintsou@nhri.edu.tw)

Table of Contents

Supporting Figure 1 ……………………………………………………………..………....2-3

Copies of ^{1}H NMR, ^{13}C NMR and mass spectra ………... 4 -27

Zn8_DM1: 0H VS. 24H in plasma.

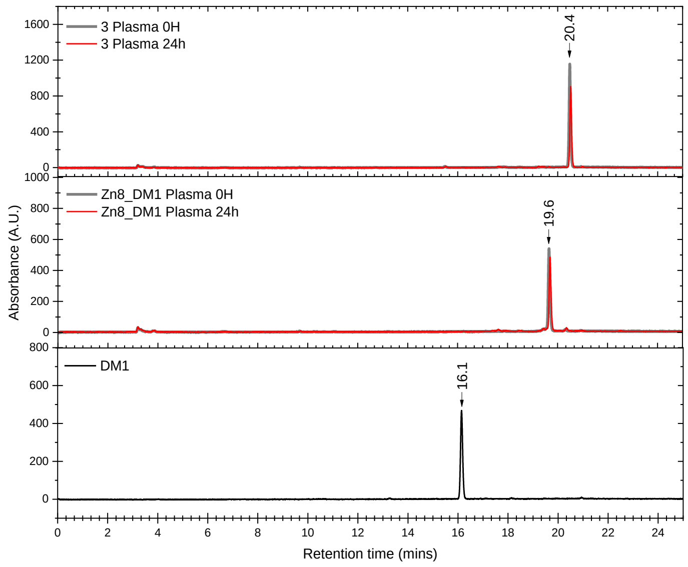

Zn10_DM4: 0H VS. 24H in plasma.

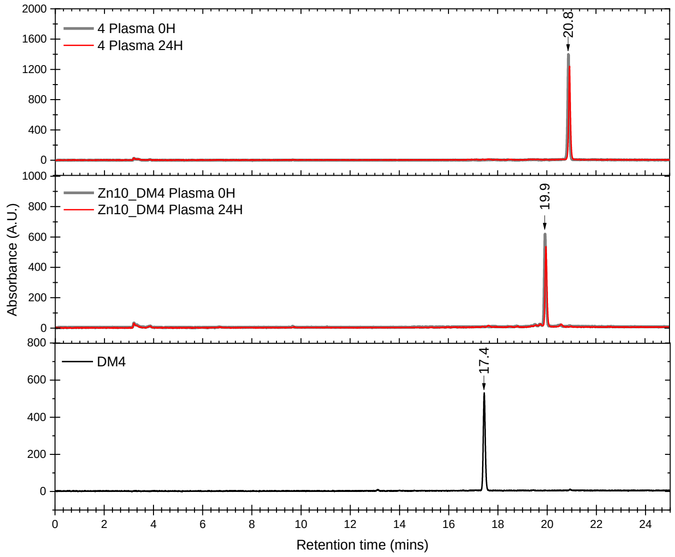

Supporting Figure S1. Incubation of Linker-drug 3 and 4, conjugates Zn8_DM1 and Zn10_DM4, DM1 and DM4 with plasma for 24 hrs. HPLC condition is listed below.

HPLC Information
Agilent 1100 HPLC was utilized in this project. Detailed chromatographic conditions used for purity analysis are listed in Table S1.

Table S1. Chromatographic conditions and parameters

Parameters	Value	
**Column**	Thermo hypersil GOLD 250*4.6 particle: 5μm	
**Mobile Phase**	A: 0.1% TFA in H_2O ; B: MeOH	
**Gradient table**	Time(min)	% B
	0	10
	2	10
	6	50
	16	90
	17	90
**Flow rate**	0.5 ml/min	
**Injection volume**	10μL	
**Detector wavelength**	UV at 254 nm	
**Run time**	25 min	
**Post time**	6 min	
**Colunm temperature**	RT	
**Sample temperature**	RT	

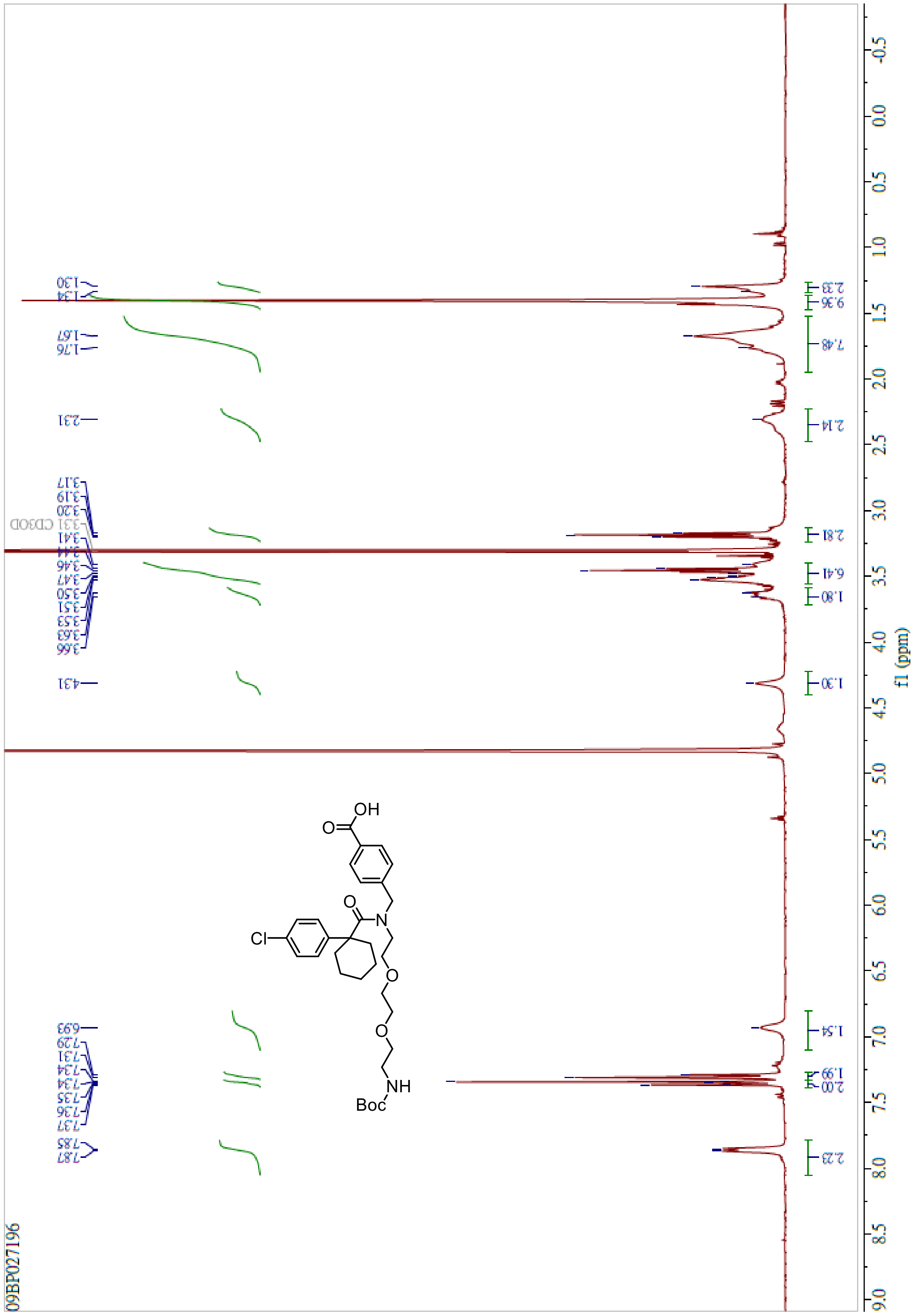
09BP027196
f1 (ppm)
3.31 CD3OD
Cl
O
N
O
O
NH
Boc
OH

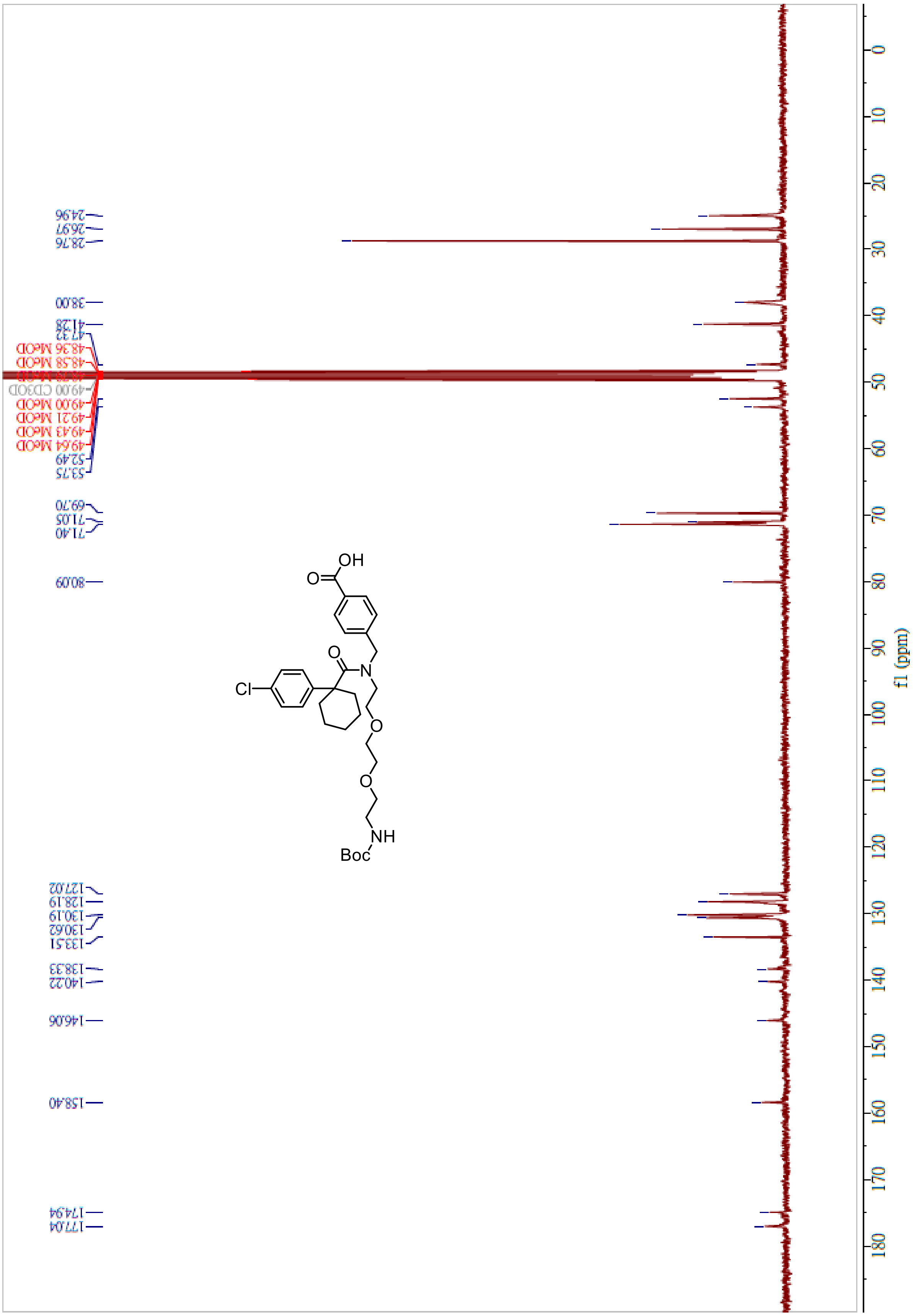
f1 (ppm)

Acquisition Parameter

Source Type	ESI	Ion Polarity	Positive	Set Nebulizer	1.0 Bar
Focus	Active	Set Capillary	4500 V	Set Dry Heater	200 °C
Scan Begin	50 m/z	Set End Plate Offset	-500 V	Set Dry Gas	6.0 l/min
Scan End	1500 m/z	Set Charging Voltage	2000 V	Set Divert Valve	Waste
		Set Corona	0 nA	Set APCI Heater	0 °C

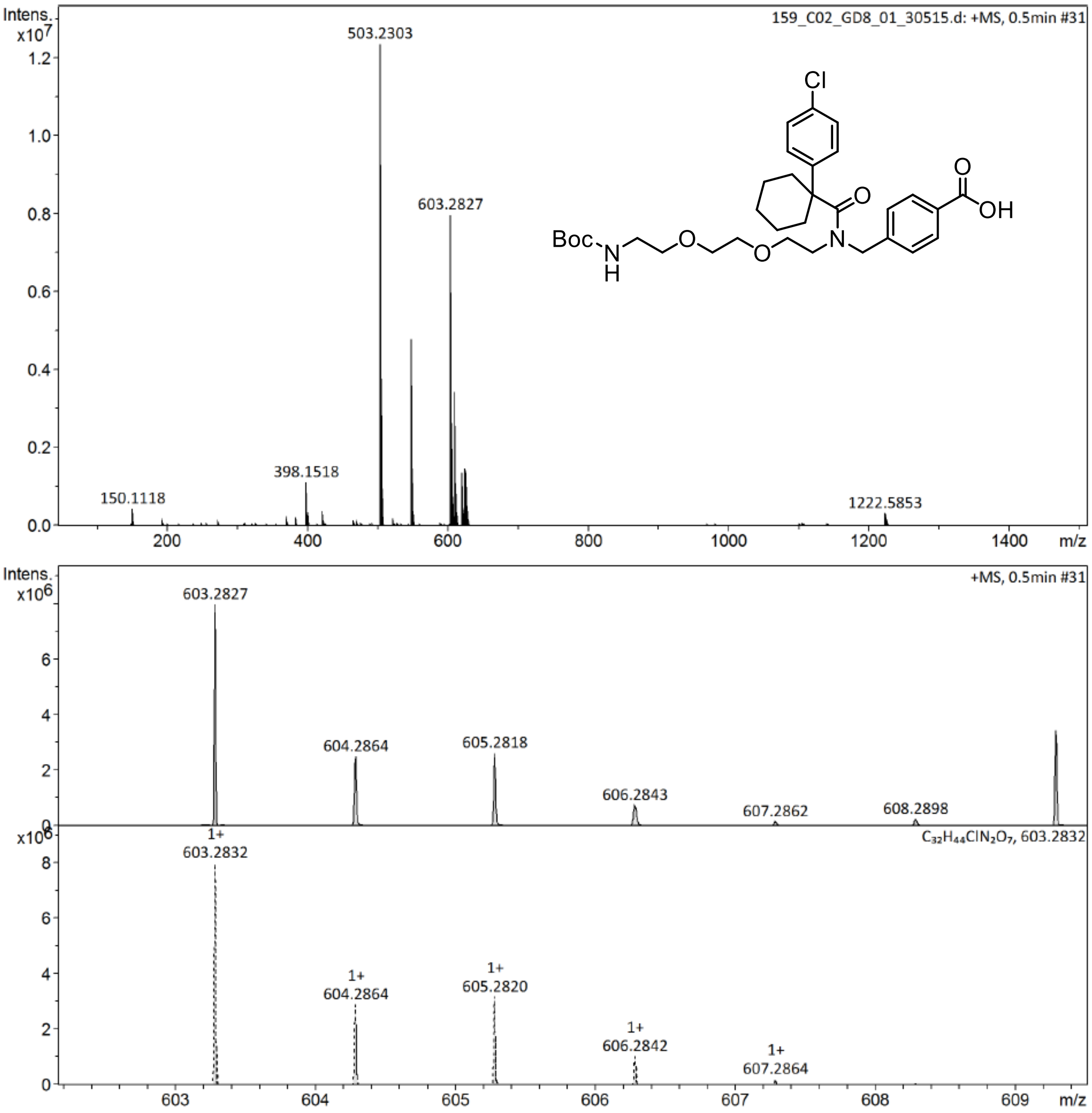

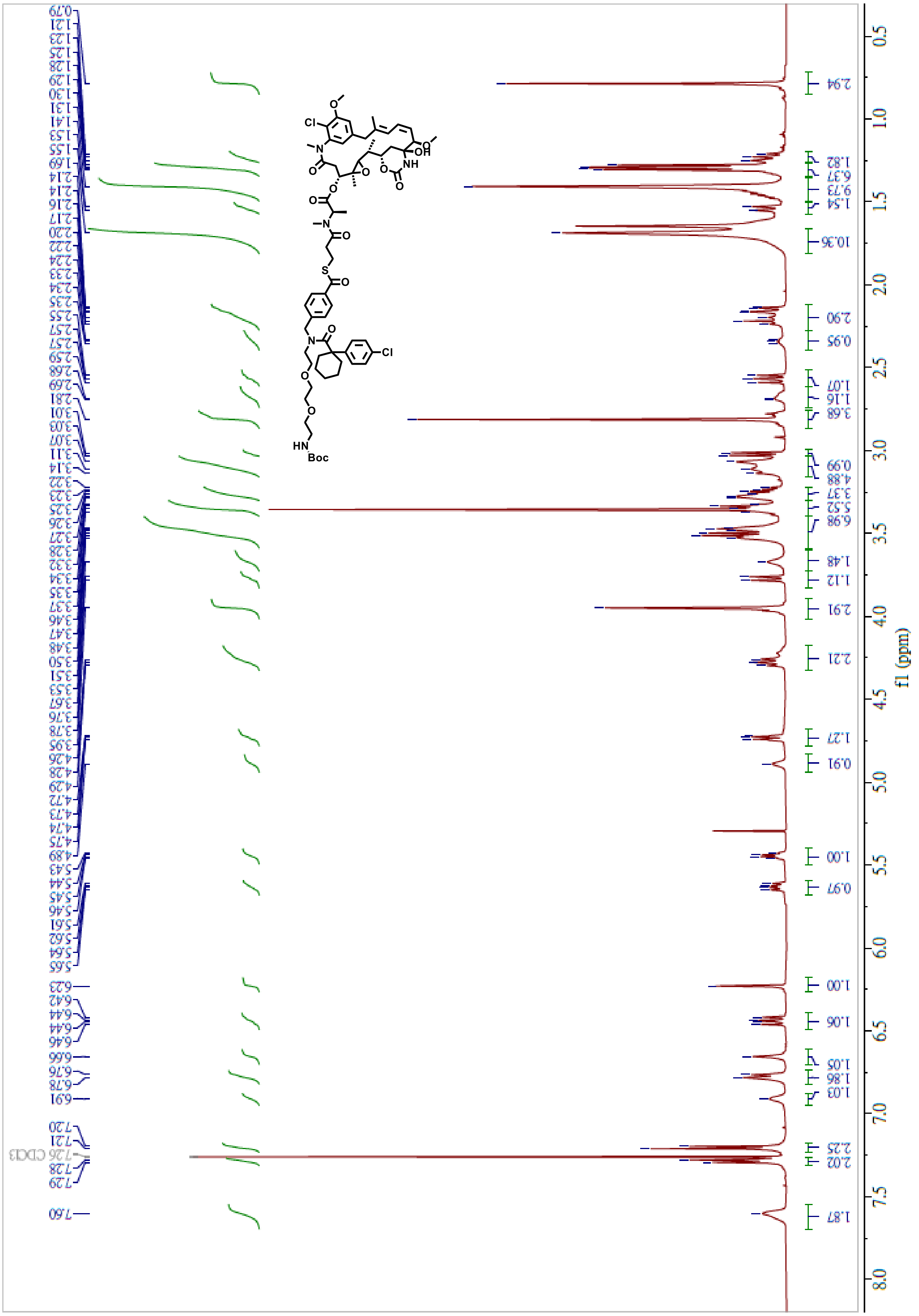
f1 (ppm)
7.26 CDCl3

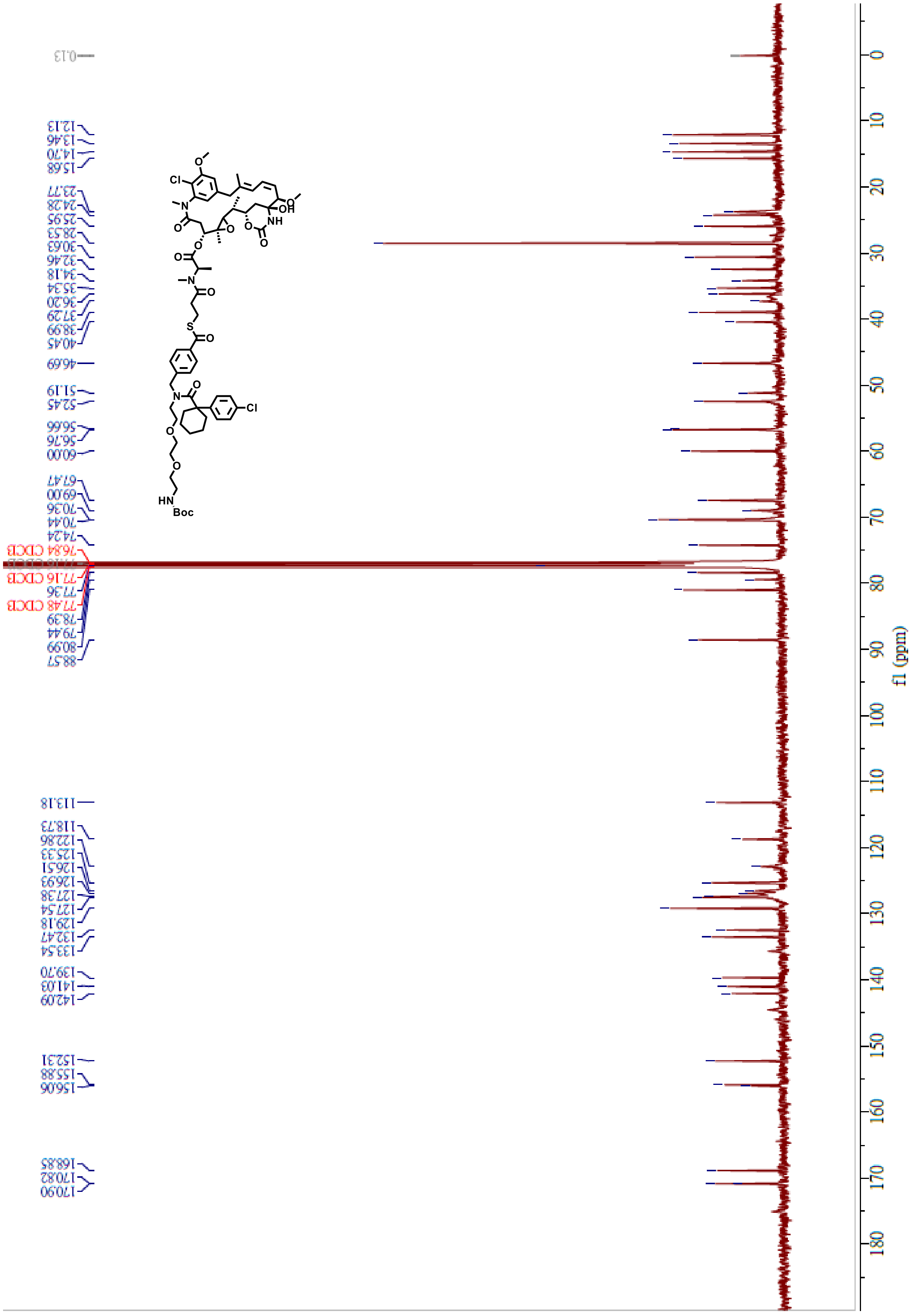

Acquisition Parameter

Source Type	ESI	Ion Polarity	Positive	Set Nebulizer	1.0 Bar
Focus	Active	Set Capillary	4500 V	Set Dry Heater	200 °C
Scan Begin	50 m/z	Set End Plate Offset	-500 V	Set Dry Gas	6.0 l/min
Scan End	1500 m/z	Set Charging Voltage	2000 V	Set Divert Valve	Waste
		Set Corona	0 nA	Set APCI Heater	0 °C

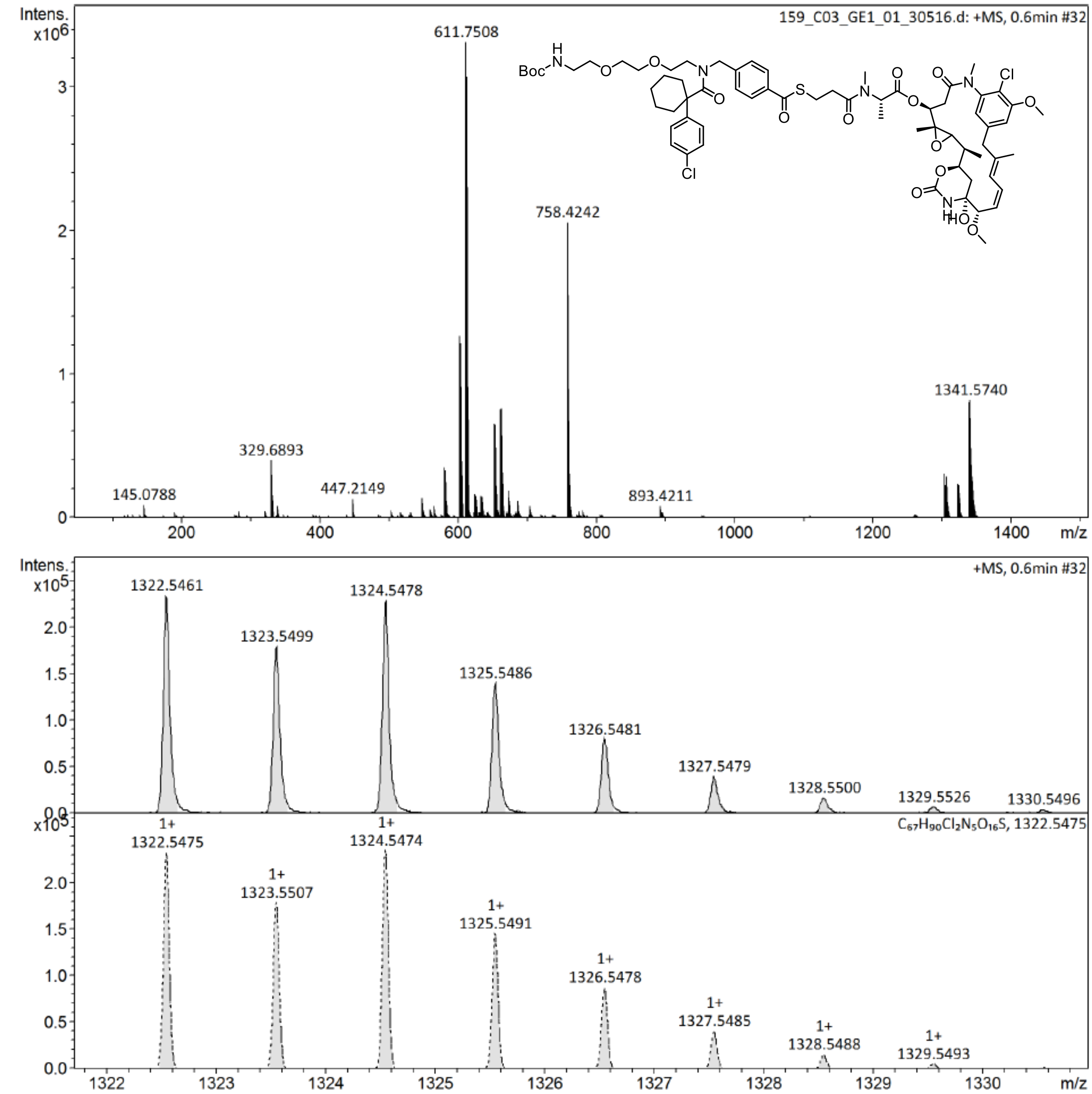

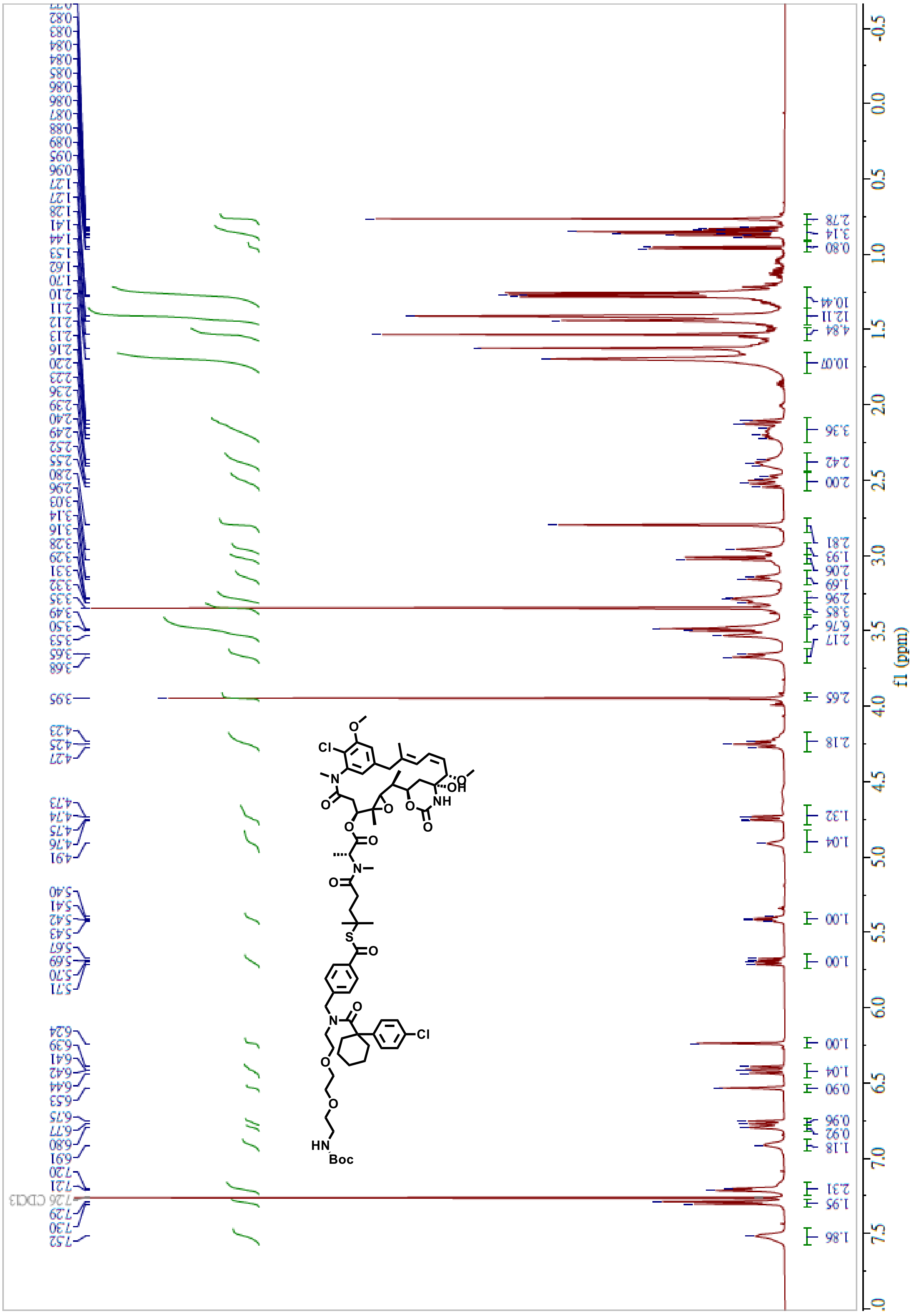
f1 (ppm)

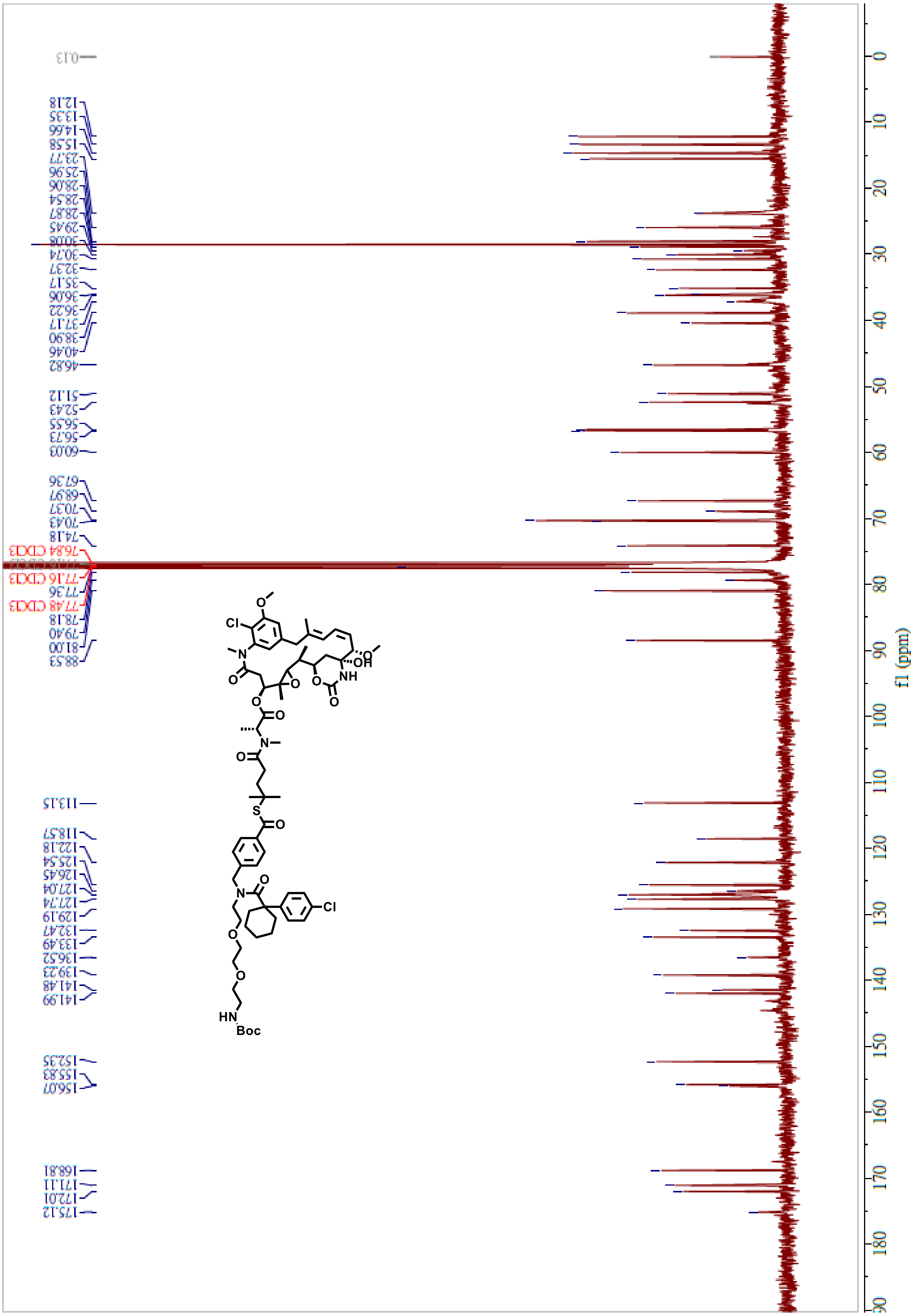
0.13
12.18
13.35
14.66
15.58
23.77
25.96
28.06
28.54
28.87
29.45
30.08
30.74
32.37
35.17
36.06
36.22
37.17
38.90
40.46
46.82
51.12
52.43
56.55
56.73
60.03
67.36
68.97
70.37
70.43
74.18
76.84 CDCl3
77.16 CDCl3
77.36
77.48 CDCl3
78.18
79.40
81.00
88.53
113.15
118.57
122.18
125.54
126.45
127.04
127.74
129.19
132.47
133.49
136.52
139.23
141.48
141.99
152.35
155.83
156.07
168.81
171.11
172.01
175.12
f1 (ppm)
Cl
OH
NH
S
HN
Boc

Acquisition Parameter

Source Type	ESI	Ion Polarity	Positive	Set Nebulizer	1.0 Bar
Focus	Active	Set Capillary	4500 V	Set Dry Heater	200 °C
Scan Begin	50 m/z	Set End Plate Offset	-500 V	Set Dry Gas	6.0 l/min
Scan End	1500 m/z	Set Charging Voltage	2000 V	Set Divert Valve	Waste
		Set Corona	0 nA	Set APCI Heater	0 °C

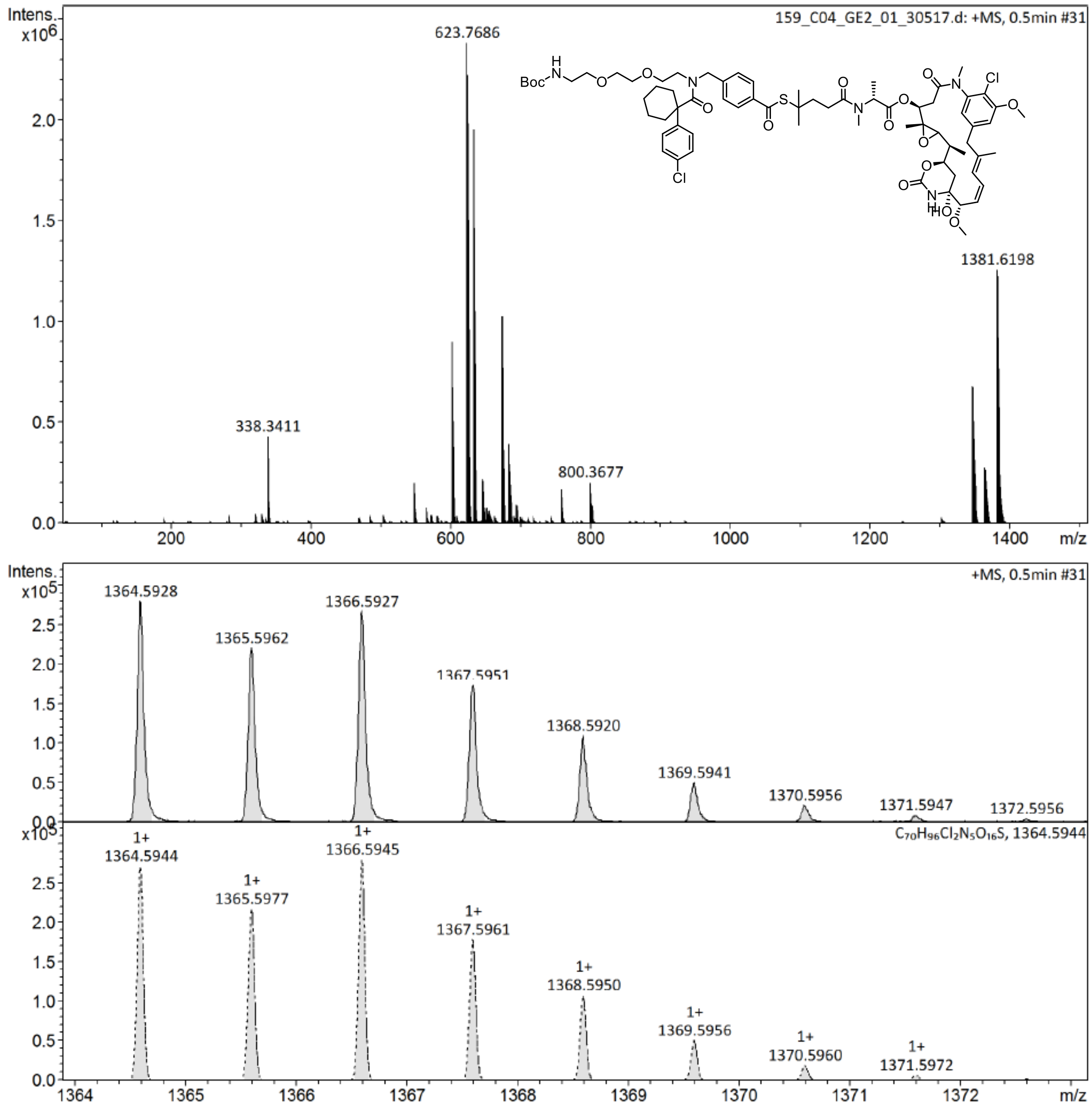

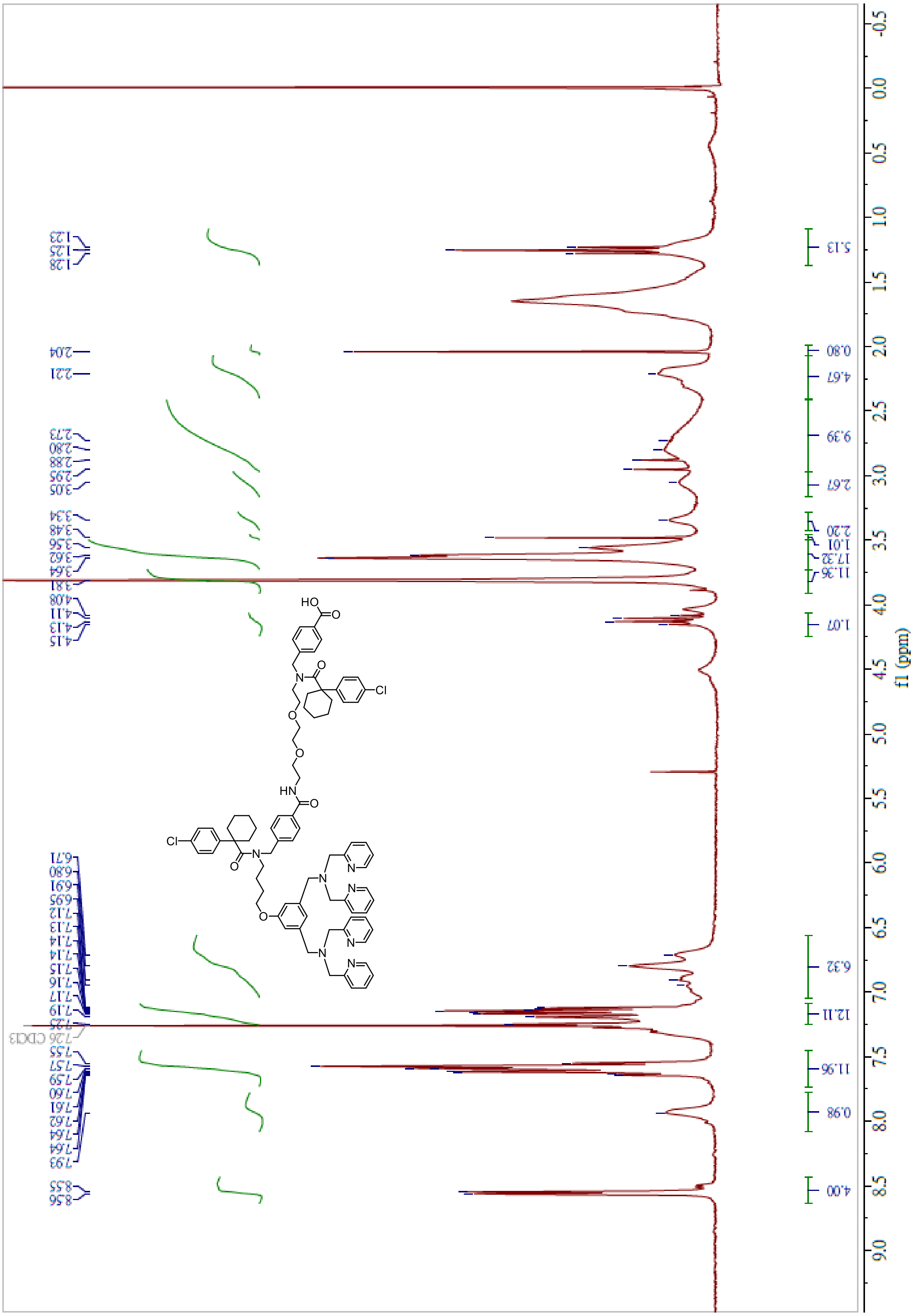

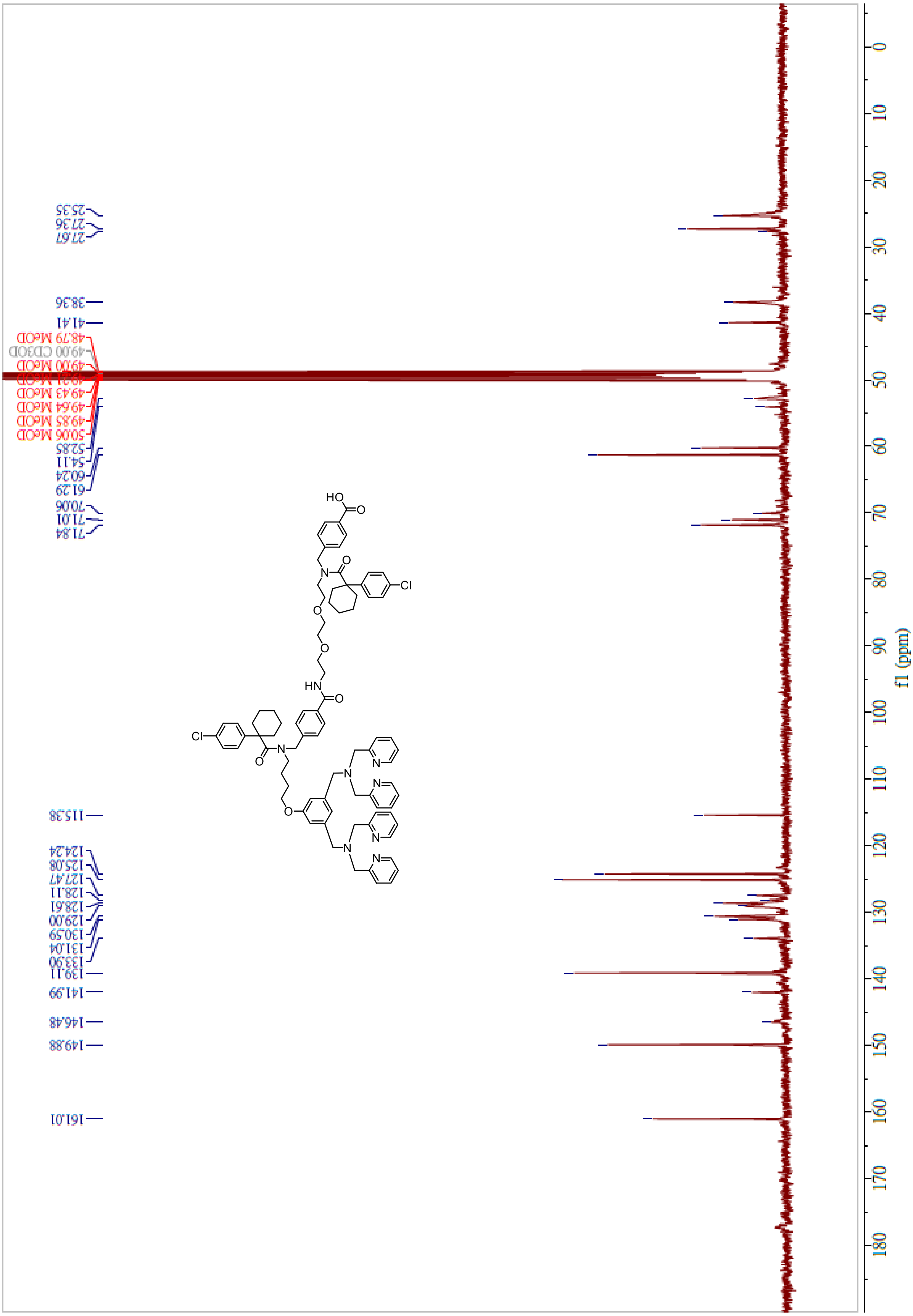

25.35
27.36
27.67
38.36
41.41
48.79 MeOD
49.00 CD3OD
49.00 MeOD
49.21 MeOD
49.43 MeOD
49.64 MeOD
49.85 MeOD
50.06 MeOD
52.85
54.11
60.24
61.29
70.06
71.01
71.84
115.38
124.24
125.08
127.47
128.11
128.61
129.00
130.59
131.04
133.90
139.11
141.99
146.48
149.88
161.01
HO
O
N
Cl
HN
f1 (ppm)

Charge number:1 Tolerance:150.00[ppm], 100.00 .. 100.... Unsaturation Number:-200.5 .. 300.0 (...
Element:^{12}C:84 .. 84, ^{1}H:0 .. 94, ^{35}Cl:2 .. 2, ^{14}N:9 .. 9, ^{23}Na:0 .. 2, ^{16}O:8 .. 8

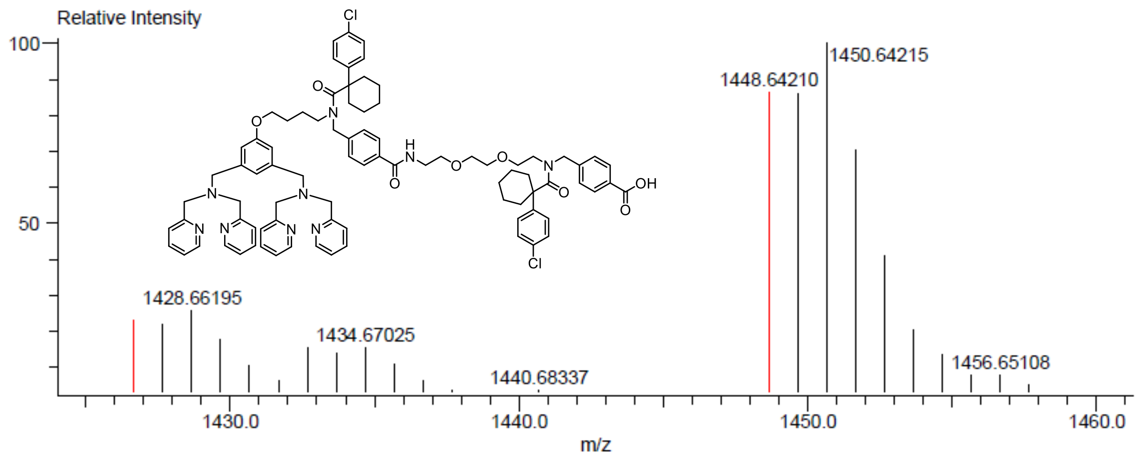

Mass	Intensity	Calc. Mass	Mass Difference [ppm]	Possible Formula
1426.66139	28257.29	1426.66024	0.81	$^{12}C_{84}{}^{1}H_{94}{}^{35}Cl_2{}^{14}N_9{}^{16}O_8$
1448.64210	106156.39	1448.64218	-0.06	$^{12}C_{84}{}^{1}H_{93}{}^{35}Cl_2{}^{14}N_9{}^{23}Na_1{}^{16}O_8$

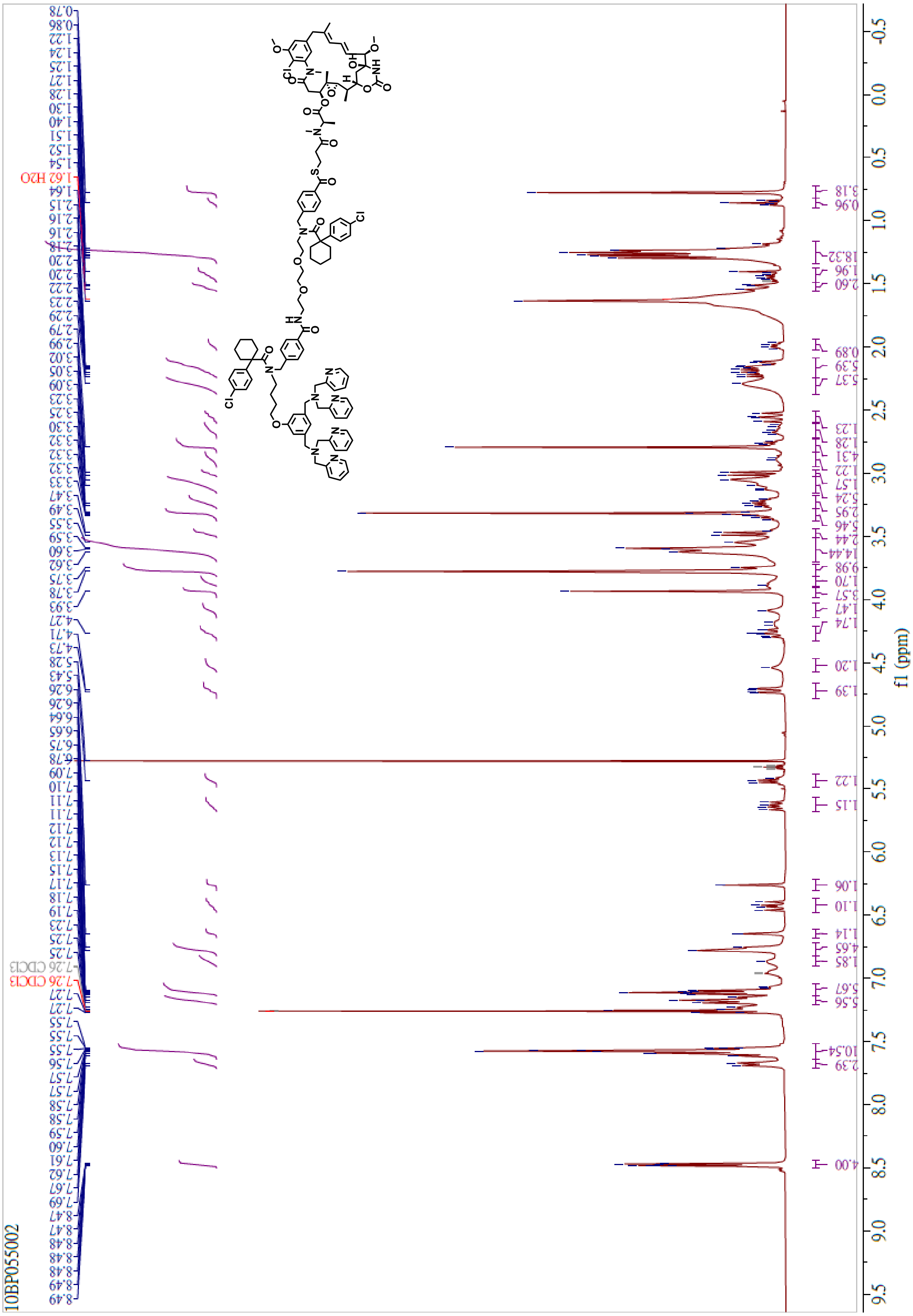
10BP055002
f1 (ppm)

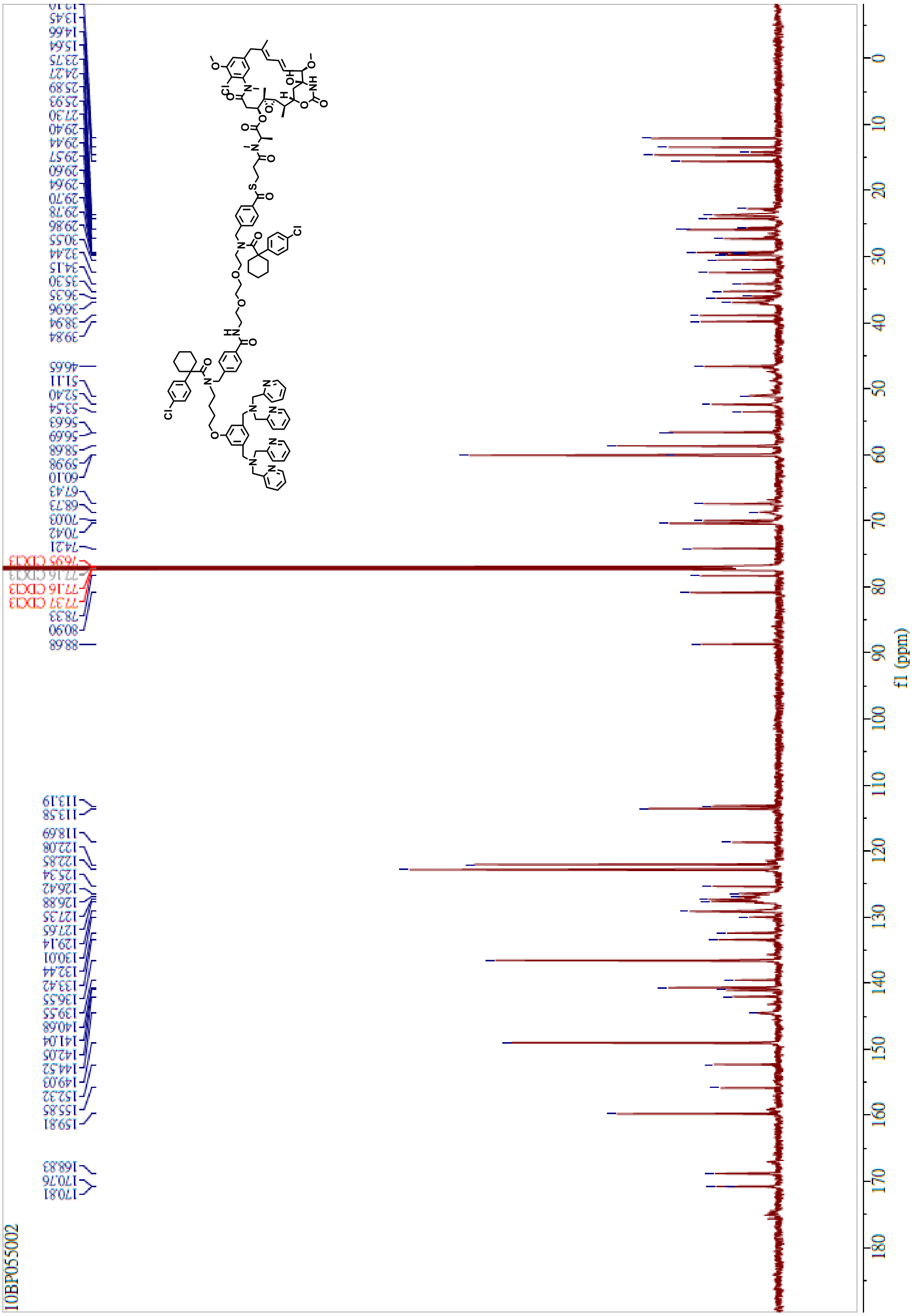
10BP055002
f1 (ppm)

Charge number:1 Tolerance:300.00[ppm], 250.00 .. 250.... Unsaturation Number:-100.0 .. 200.0 (...

Element:^{12}C:119 .. 119, ^{1}H:0 .. 140, ^{35}Cl:3 .. 3, ^{14}N:12 .. 12, ^{23}Na:0 .. 1, ^{16}O:17 .. 17, ^{32}S:1 .. 1

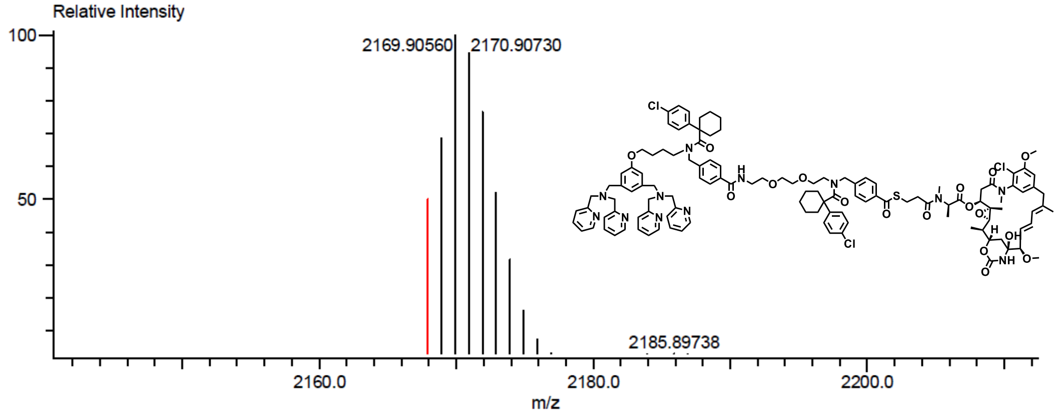

Mass	Intensity	Calc. Mass	Mass Difference [mDa]	Mass Difference [ppm]
2167.90420	54324.13	2167.90651	-2.31	-1.06

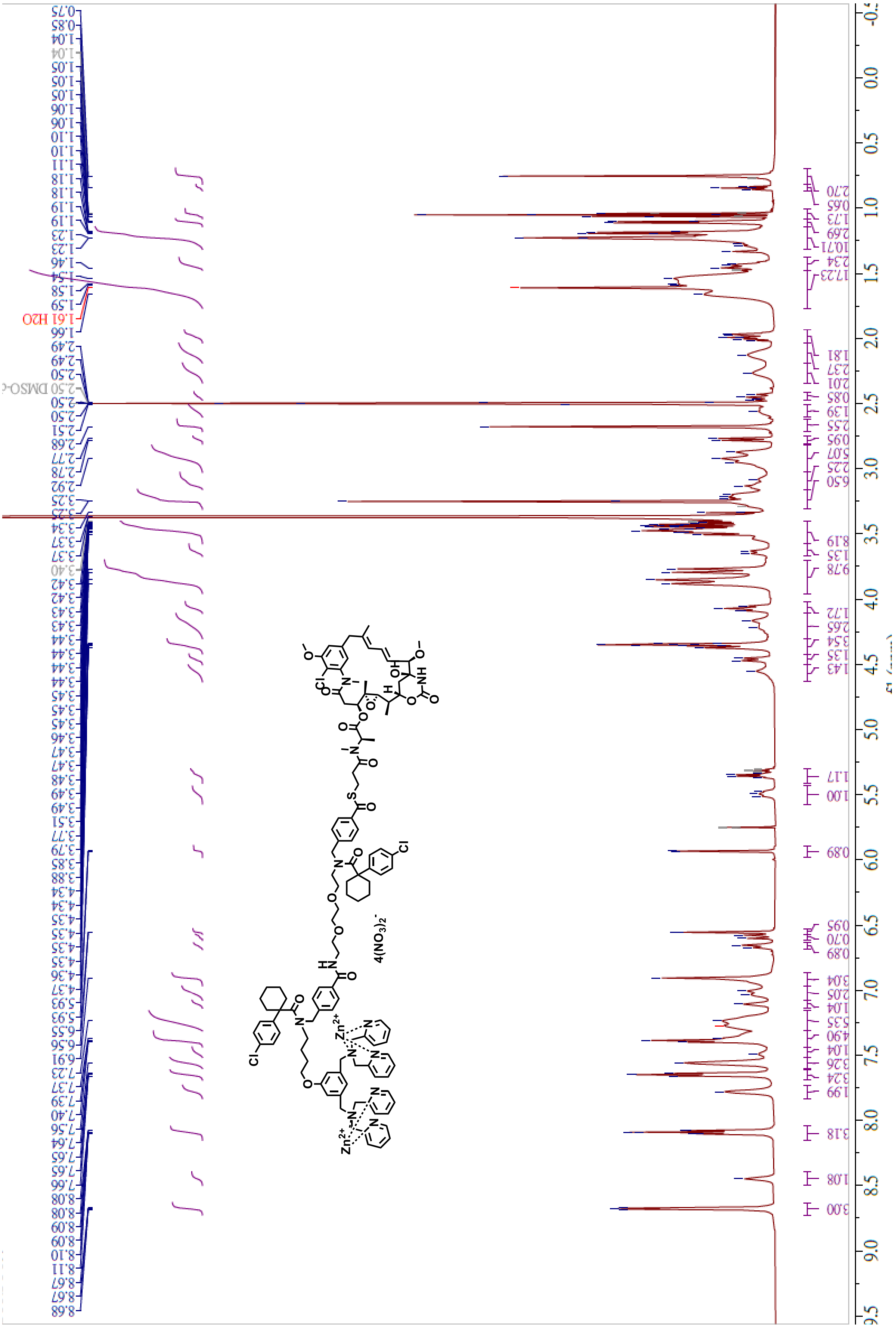

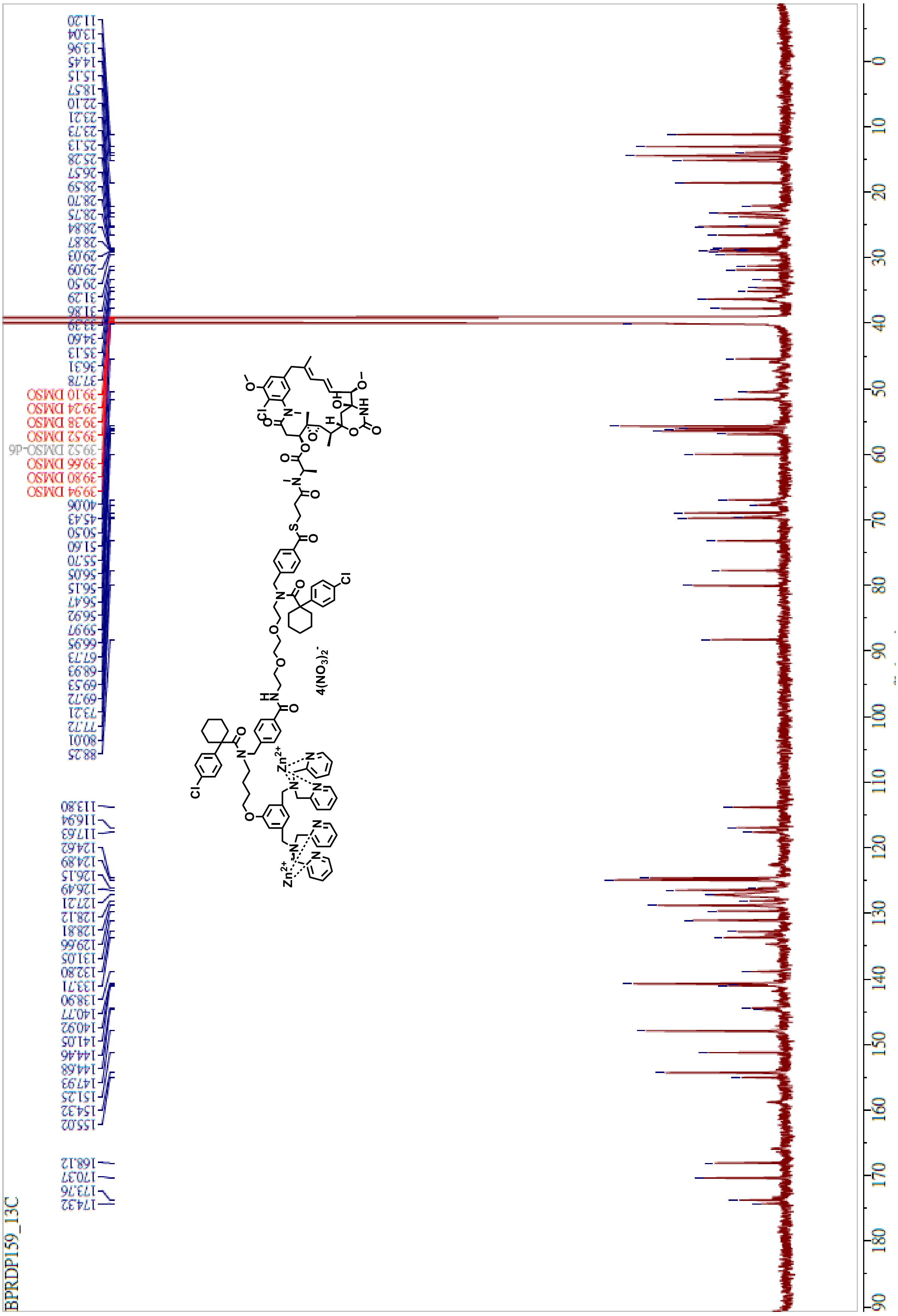
BPRDP159_13C
174.32
173.76
170.37
168.12
155.02
154.32
151.25
147.93
144.68
144.46
141.05
140.92
140.77
138.90
133.71
132.80
131.05
129.66
128.81
128.12
127.21
126.49
126.15
124.89
124.62
117.63
116.94
113.80
88.25
80.01
77.72
73.21
69.72
69.53
68.93
67.73
66.95
59.97
56.92
56.47
56.15
56.05
55.70
51.60
50.50
45.43
40.06
39.94 DMSO
39.80 DMSO
39.66 DMSO
39.52 DMSO-d6
39.38 DMSO
39.24 DMSO
39.10 DMSO
37.78
36.31
35.13
34.60
31.86
31.29
29.50
29.09
29.03
28.87
28.84
28.75
28.70
28.59
26.57
25.28
25.13
23.73
23.21
22.10
18.57
15.15
14.45
13.96
13.04
11.20
4(NO3)2-
190
180
170
160
150
140
130
120
110
100
90
80
70
60
50
40
30
20
10
0

Acquisition Parameter

Source Type	ESI	Ion Polarity	Positive	Set Nebulizer	1.0 Bar
Focus	Active	Set Capillary	4500 V	Set Dry Heater	200 °C
Scan Begin	50 m/z	Set End Plate Offset	-500 V	Set Dry Gas	6.0 l/min
Scan End	3000 m/z	Set Charging Voltage	2000 V	Set Divert Valve	Waste
		Set Corona	0 nA	Set APCI Heater	0 °C

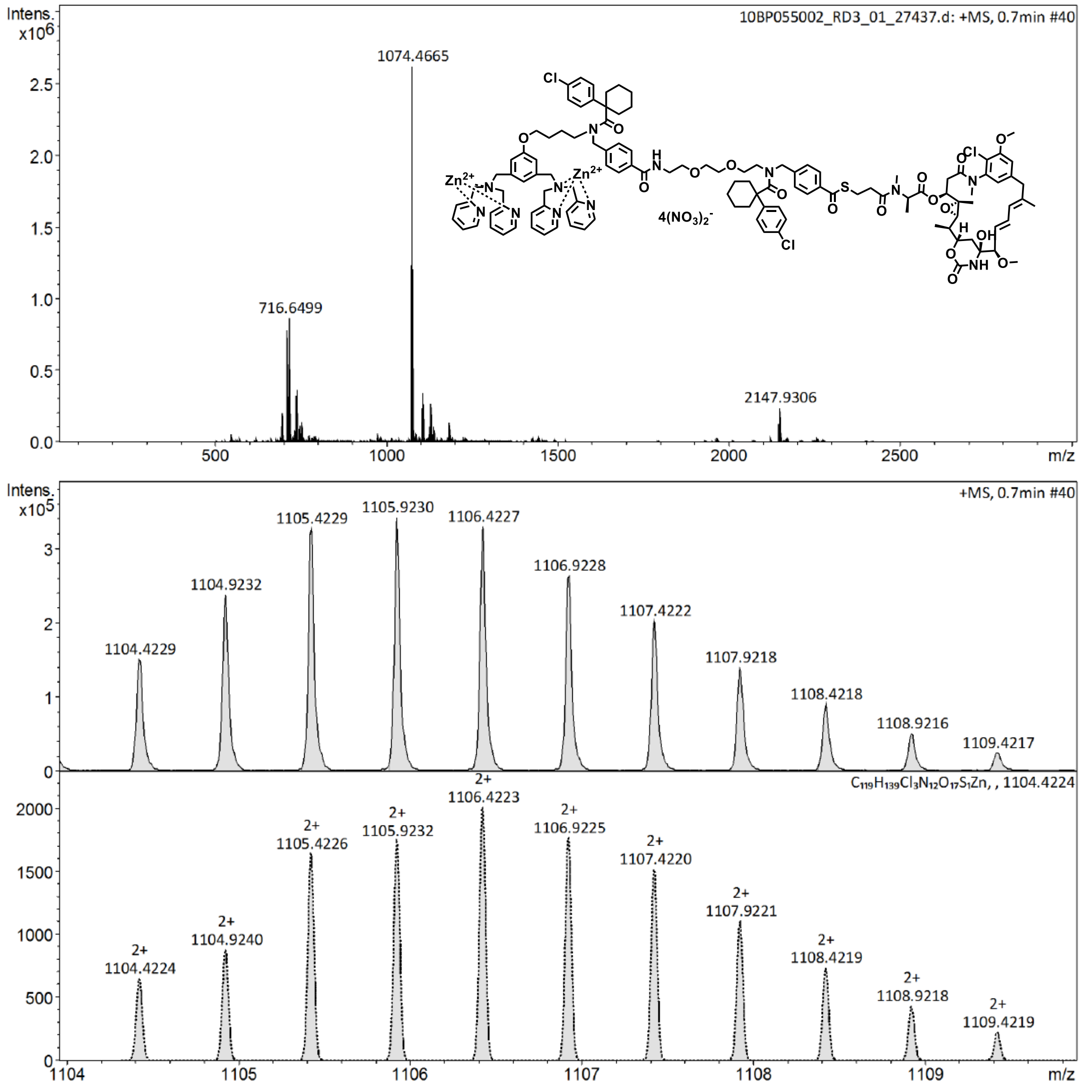

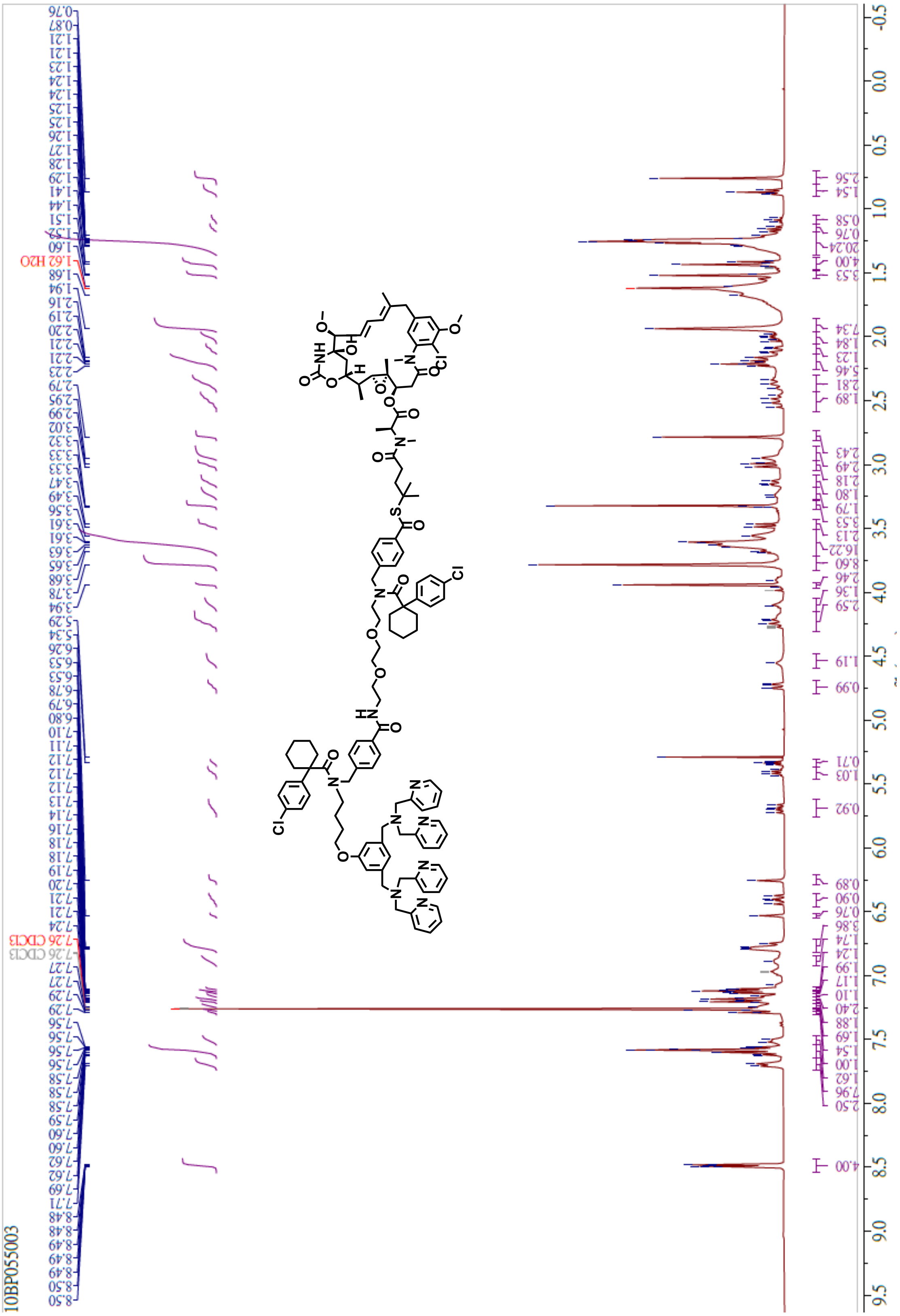
10BP055003

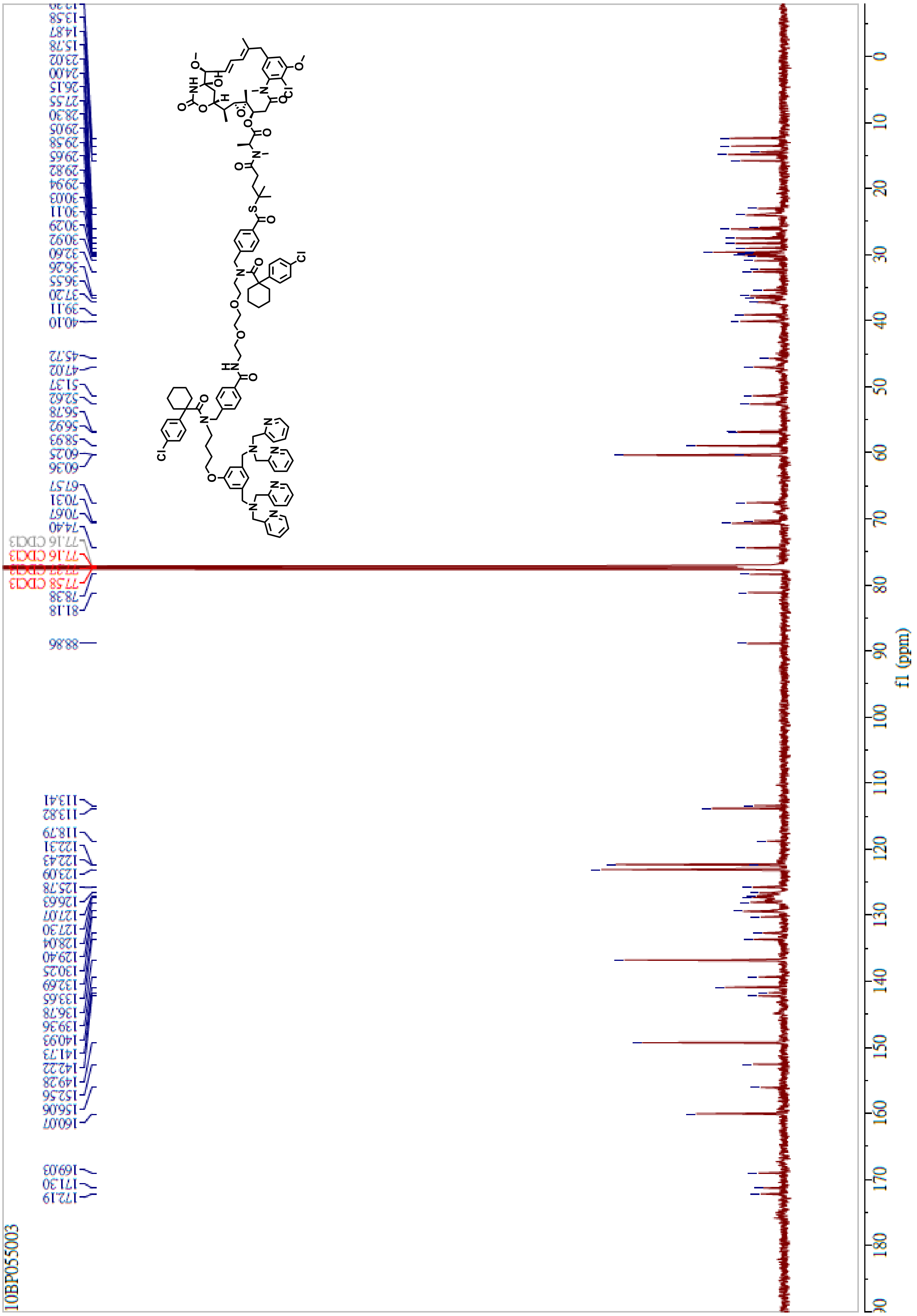
10BP055003
f1 (ppm)
13.58
14.87
15.78
23.02
24.00
26.15
27.55
28.30
29.05
29.58
29.65
29.82
29.94
30.03
30.11
30.29
30.92
32.60
36.26
36.55
37.20
39.11
40.10
45.72
47.02
51.37
52.62
56.78
56.92
58.93
60.25
60.36
67.57
70.31
70.67
74.40
77.16 CDCl3
77.16 CDCl3
77.58 CDCl3
78.38
81.18
88.86
113.41
113.82
118.79
122.31
122.43
123.09
125.78
126.63
127.07
127.30
128.04
129.40
130.25
132.69
133.65
136.78
139.36
140.93
141.73
142.22
149.28
152.56
156.06
160.07
169.03
171.30
172.19

Charge number:1 Tolerance:300.00[ppm], 250.00 .. 250.... Unsaturation Number:-100.0 .. 200.0 (...
Element:^{12}C:122 .. 122, ^{1}H:0 .. 146, ^{35}Cl:3 .. 3, ^{14}N:12 .. 12, ^{23}Na:0 .. 1, ^{16}O:17 .. 17, ^{32}S:1 .. 1

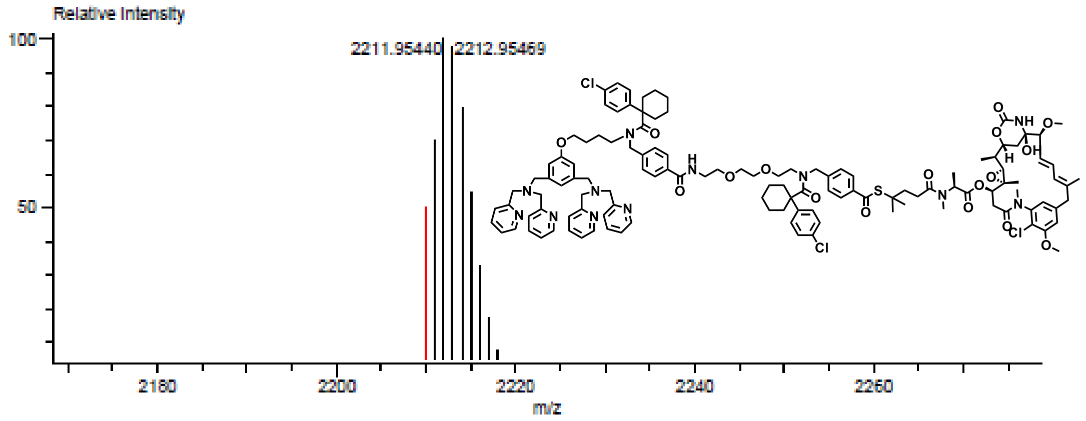

Mass	Intensity	Calc. Mass	Mass Difference [mDa]	Mass Difference [ppm]
2209.95189	25511.96	2209.95346	-1.57	-0.71

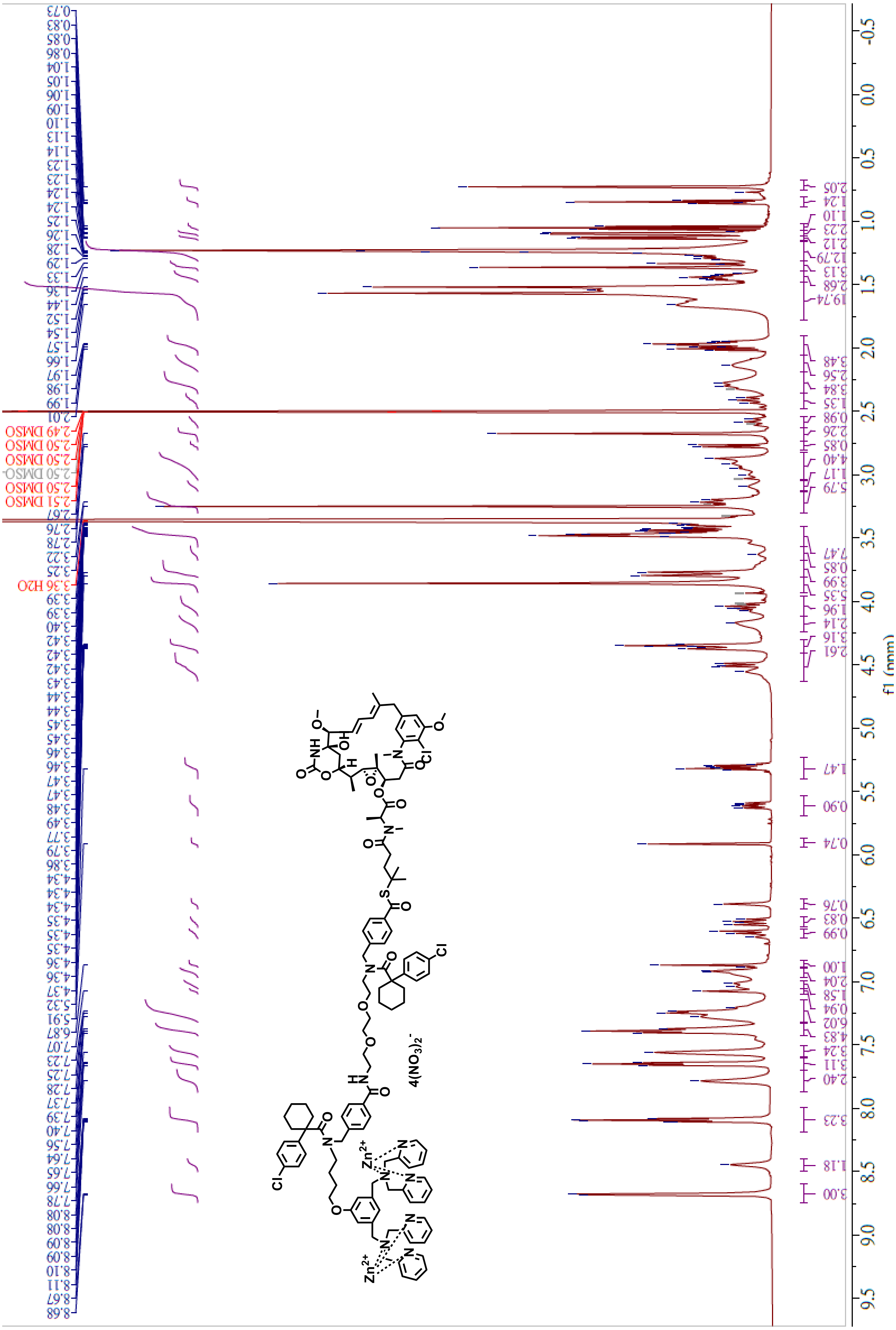

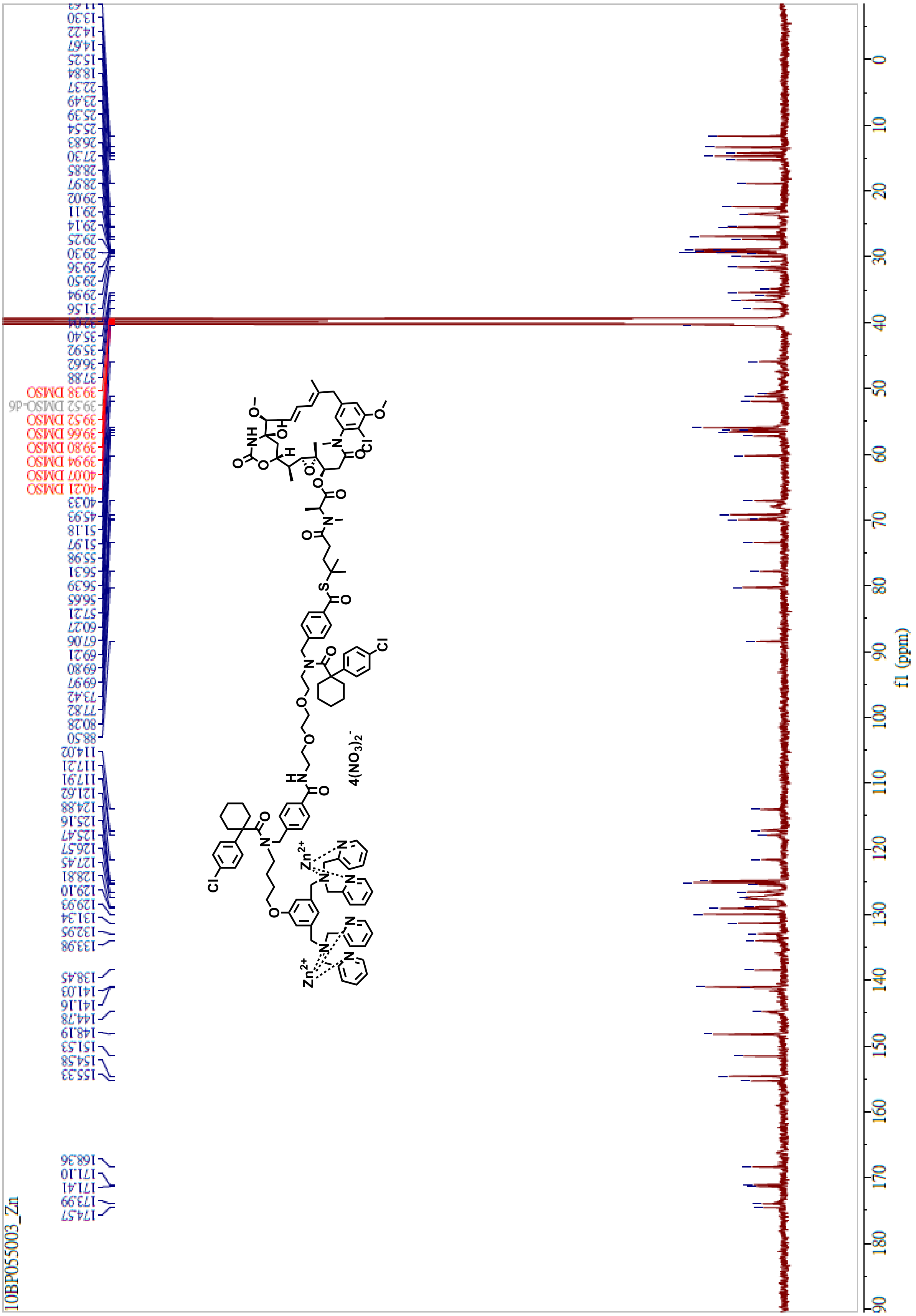
10BP055003_Zn
f1 (ppm)

Acquisition Parameter

Source Type	ESI	Ion Polarity	Positive	Set Nebulizer	1.0 Bar
Focus	Active	Set Capillary	4500 V	Set Dry Heater	200 °C
Scan Begin	50 m/z	Set End Plate Offset	-500 V	Set Dry Gas	6.0 l/min
Scan End	3000 m/z	Set Charging Voltage	2000 V	Set Divert Valve	Waste
		Set Corona	0 nA	Set APCI Heater	0 °C

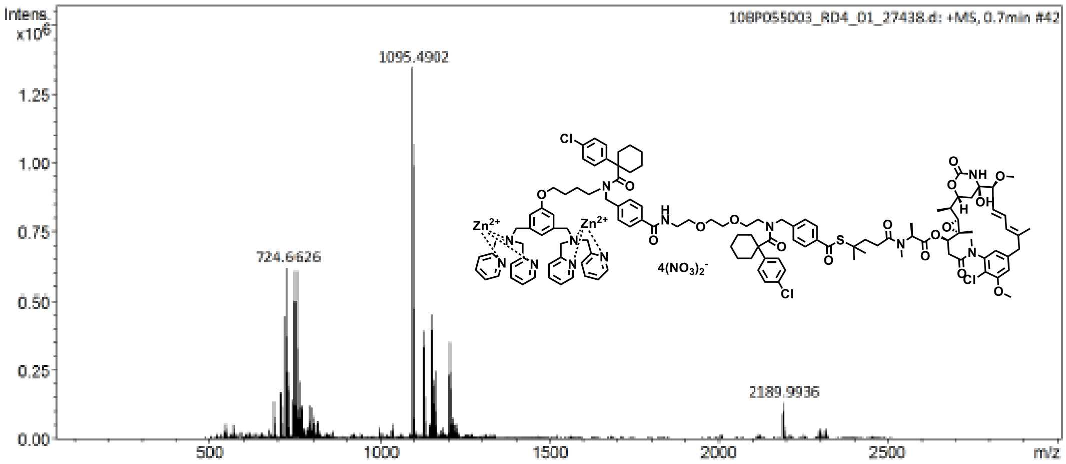

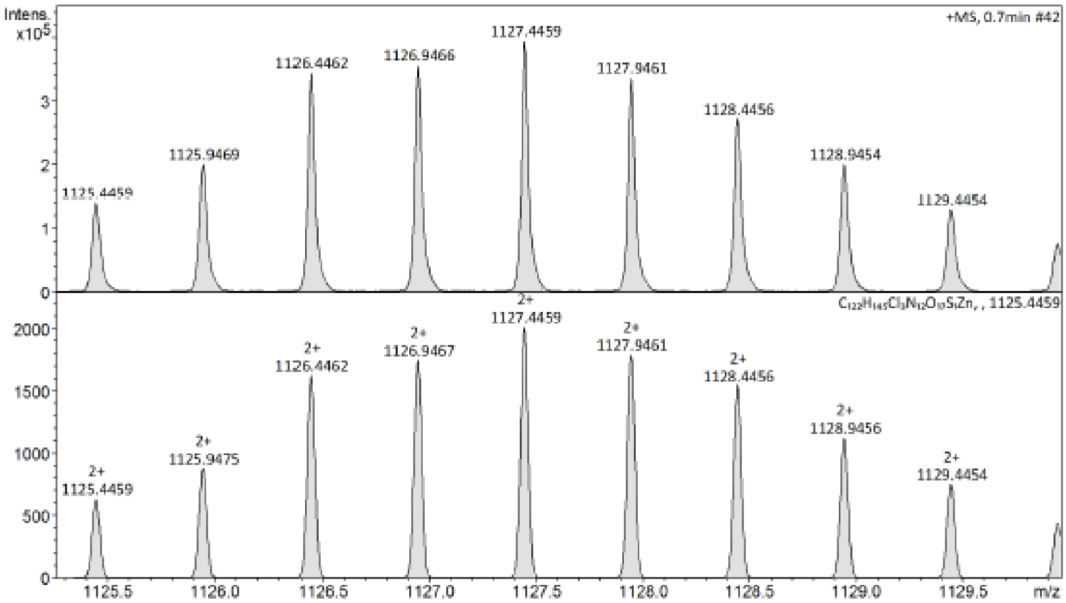

Supplement: Supplementary file 1 [file pharmaceutics-14-01316-s001.zip › pharmaceutics-1755407-supplementary.pdf]
